# Analysing open-source protein folding models for nanobody binding prediction

**DOI:** 10.3389/fbinf.2026.1858891

**Published:** 2026-07-22

**Authors:** Yannick Vogt, Rebekka Roßberg, Jan Habermann, Pia Kluge, Wendan Xu, Mehdi Naouar, Stefan Hirschberg, Cornelius Miething, Joschka Bödecker, Maria Kalweit, Evelyn Ullrich, Gabriel Kalweit

**Affiliations:** 1 Department of Computer Science, University of Freiburg, Freiburg, Germany; 2 Collaborative Research Institute Intelligent Oncology (CRIION), Freiburg, Germany; 3 Experimental Immunology and Cell Therapy, Department of Pediatrics, Goethe University, Frankfurt am Main, Germany; 4 Department of Medicine I, University Medical Center Freiburg, University of Freiburg, Freiburg, Germany; 5 German Cancer Consortium (DKTK), Partner Site Freiburg, Freiburg, Germany; 6 preclinics certified products GmbH, Potsdam, Germany; 7 preclinics Gesellschaft für präklinische Forschung mbH, Potsdam, Germany; 8 Intelligent Machine-Brain Interfacing Technology (IMBIT), BrainLinks-BrainTools, University of Freiburg, Freiburg, Germany; 9 German Cancer Consortium (DKTK), Partner Site Frankfurt/Mainz, Frankfurt am Main, Germany

**Keywords:** AlphaFold3, binding prediction, Boltz-2, Chai-1, confidence measure, in silico screening, nanobody, IntFold

## Abstract

**Introduction:**

Antibody-based therapeutics are a rapidly expanding class of treatments, with over 200 approved candidates and thousands in clinical trials. Computational pre-filtering using protein structure prediction models has the potential to reduce the cost of wet-lab screening, yet the relationship between model confidence measures and functional binding properties remains incompletely understood. Here, we evaluate whether confidence measures produced by contemporary open-source protein structure prediction models are suitable for *in silico* screening of nanobody–antigen interactions.

**Methods:**

We benchmark Boltz-2, Chai-1, IntFold, and AlphaFold3 using two complementary tasks: (i) ranking true nanobody–antigen binding complexes above non-binding bait pairs across 17 antigens, and (ii) detecting out-of-distribution sequences generated by alanine substitution of all complementarity-determining region residues. We further assess confidence measure sensitivity through progressive alanine mutagenesis on 13 nanobody–antigen complexes spanning the range of CDR3 lengths in our dataset and evaluate generalizability using data from a camelid immunization campaign against CD33.

**Results:**

Boltz-2-derived confidence measures achieved the highest median performance for identifying true binders. Local confidence measures, including pLDDT and interface- or CDR-focused metrics, were most effective at detecting out-of-distribution sequences and exhibited the greatest sensitivity to mutations. No single confidence measure performed best across both tasks, and all evaluated protein structure prediction models showed limited generalization to previously unseen antigens.

**Discussion:**

Our results suggest that robust *in silico* nanobody candidate selection should combine complementary global and local confidence measures rather than relying on a single metric. These findings provide practical guidance for integrating open-source protein structure prediction models into AI-driven nanobody discovery pipelines while highlighting the need for improved generalization across antigens.

## 1 Introduction

**FIGURE 1:**
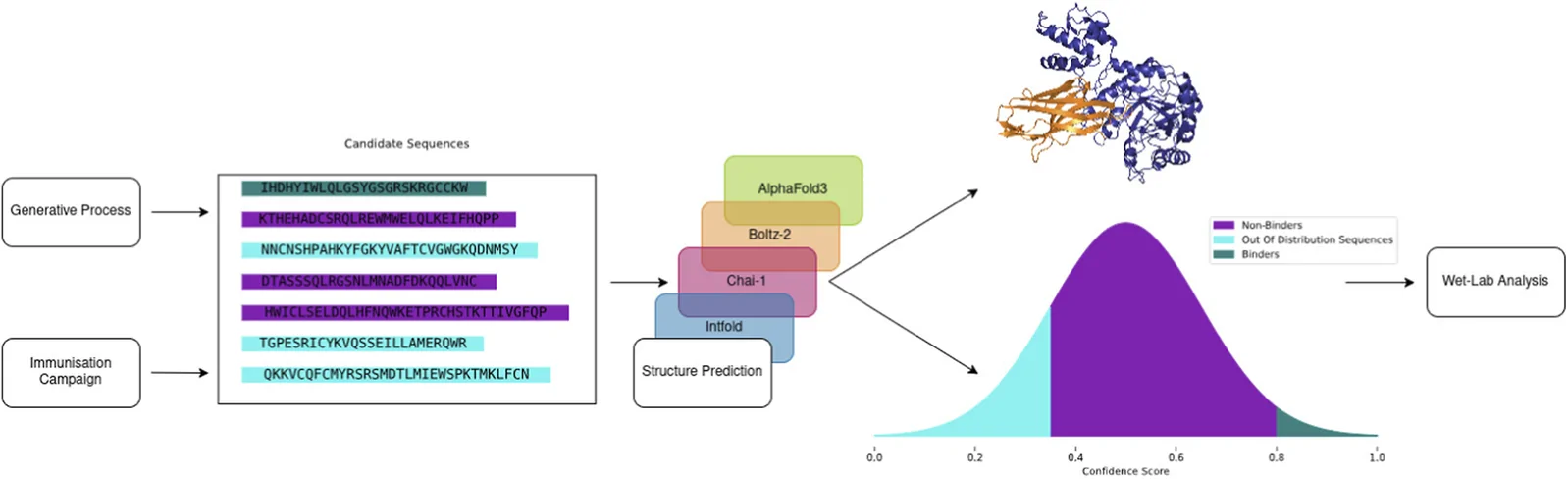
Overview of antibody design using structure-prediction based filtering: We start from a list of candidate antibody sequences for binding an antigen of interest, derived, e.g., from immunisation campaigns or generative processes. This list of candidates might contain binders, non-binders, and non-functioning OOD sequences. To reduce the number of sequences tested in cost-intensive wet-lab analysis, candidates can be pre-filtered using confidence measures predicted by structure prediction models. A perfect measure would thereby separate binders from non-binding and OOD sequences, leaving only the most promising candidates for real-world analysis.

Therapeutic antibodies offer substantial potential for treating a broad range of diseases, including a wide range of cancer types. Yet the identification and validation of binding candidates through traditional wet-lab experimentation is constrained by high costs and long timelines. Computational approaches, including generative AI, have demonstrated considerable promise for candidate design, but evaluating the output of such methods remains expensive. Effective *in silico* pre-filtering, as illustrated in Figure 1, is therefore of great practical importance. A range of methods have been developed for this purpose, from sequence-level affinity predictors to structure-based scoring functions that consider the full quaternary complex (Abbasi et al., 2020; Romero-Molina et al., 2022; Vangone and Bonvin, 2017; Yang et al., 2023). The great impact of such pre-filtering was shown, among others, by Bennett et al. (2023) who demonstrated an increase in experimental success rates by up to tenfold. It is now common practice to apply structure prediction models in candidate pre-filtering for *in vitro* (Vázquez Torres et al., 2025; Dauparas et al., 2022; Wang et al., 2022; Jendrusch et al., 2025) experiments and as evaluation tools for *in silico* settings (Watson et al., 2023; Jendrusch et al., 2025; Melnyk et al., 2025; Kosugi and Ohue, 2022).

However, protein structure prediction methods are not without limitations. AF2 (Jumper et al., 2021) and its successor AF3 (Abramson et al., 2024) have been shown to poorly capture the effects of missense mutations (Buel and Walters, 2022; Pak et al., 2021), and to struggle with antibody–antigen complex prediction (Abramson et al., 2024), as well as nanobody docking (Hitawala and Gray, 2025). Although some analyses have addressed confidence measures for protein interaction (Bryant et al., 2022; Teufel et al., 2023), the majority of benchmarking studies focus on structural accuracy rather than binding prediction (Xu et al., 2025; Abramson et al., 2024; Passaro et al., 2025), are largely restricted to AF2, AlphaFold-Multimer (AF-M), and AF3 (Yin and Pierce, 2024; Bellinzona et al., 2024; Zhu et al., 2023), and rarely include statistical significance tests.

In recent years, several open-source structure prediction models have emerged as viable alternatives to AlphaFold models, including Boltz-1 and its successor Boltz-2 (Wohlwend et al., 2024; Passaro et al., 2025), Chai-1 (Chai Discovery et al., 2024), and IntFold (The IntFold Team et al., 2025). These models offer additional practical advantages such as permissive licensing, lower computational requirements, and reduced or no dependence on Multiple Sequence Alignment (MSA) computation. A systematic analysis of these alternatives for nanobody–antigen binding prediction is, however, still missing.

When using a protein structure prediction model for candidate evaluation, a confidence measure should rank candidates that actually bind a target antigen above those that do not. In a screening setting, such ranking serves to reduce a large candidate pool to a smaller subset enriched for binders, of which only a limited number can be advanced to costly experimental validation. Prior studies have typically evaluated this capability by comparing scores for a known binder against a panel of candidates known to bind other targets (Teufel et al., 2023; Bryant et al., 2022). While this is a valid experimental design, it considers only naturally occurring or biophysically realistic sequences. In practice, generative algorithms can produce candidates outside their training distribution. These sequences might be implausible, not because they bind a different antigen, but because they are structurally or chemically nonsensical. Detecting such OOD sequences is therefore a distinct but important capability, which has not been addressed in prior benchmarking of structure-based measures.

We focus specifically on nanobodies, consisting of the variable domains (VHH) of heavy-chain-only antibodies, which offer several favourable properties: compact size, elevated thermal stability and solubility, and an elongated CDR3 region that can access cryptic epitopes inaccessible to conventional antibodies. These characteristics make nanobodies attractive for AI-driven therapeutic design, yet they have not been explicitly considered in prior analyses of structure prediction models for *in silico* screening.

To address these gaps, we compiled a test set of 28 nanobodies targeting 17 antigens absent from the training data of all evaluated models, and constructed additional datasets of progressively or fully mutated CDR sequences. Using these, we evaluated confidence measure performance on two tasks: identifying true binders and detecting OOD sequences. Furthermore, to better understand the effects in the second task, we performed an alanine mutation sensitivity analysis assessing confidence measure responsiveness to incremental structural perturbation. Lastly, we analysed the generalisation of our results on a set of nanobody sequences derived from a camelid immunisation campaign against CD33.

Our analysis reveals that many confidence measures perform well on one task but poorly on the other, with only very few achieving competitive performance on both. Among the models evaluated, Boltz-2 consistently achieves the highest median Receiver Operating Characteristic Area Under the Curve (ROC AUC) for true binder identification, though the single best-performing measures come from IntFold and AF3, while AF3 significantly outperforms other models in OOD sequence detection. However, we find that reliable generalisation to unseen antigens remains limited.

## 2 Materials and methods

### 2.1 Datasets

Evaluating protein structure prediction method applicability for scoring previously unseen nanobody–antigen complexes requires a test set of complexes outside the training data of all evaluated models.

To construct such a dataset, we extracted all entries from the RCSB Protein Data Bank (Burley et al., 2021) published after 1 June 2023, that contain a nanobody in contact with an antigen. This cutoff date excludes all data on which any of the evaluated methods were trained (Abramson et al., 2024; The IntFold Team et al., 2025; Passaro et al., 2025; Chai Discovery et al., 2024). We then grouped entries by target antigen using a 95% sequence similarity cutoff and retained only those with no known binders deposited in the RCSB before the cutoff date, ensuring that no evaluated model was trained on any of the included antigens in a bound state and no similar antigens would bias our analysis. After removing non-parsable complexes, the resulting dataset comprises 17 antigen structures, each with confirmed binding to one or more of the 28 nanobodies. For antigens represented by multiple non-identical sequences, we selected the sequence corresponding to the structure with the highest similarity to all other deposited structures for that antigen. All antigens were modelled as protein sequence only, as extracted from the respective RCSB entries, without any co-factors or modifications.

Building on this core dataset, we constructed three complementary datasets suited to our analysis.

Identifying real binders. The core dataset yields 28 experimentally verified binding complexes and 482 bait pairings in which each nanobody is paired with an antigen it is not known to bind. We treat these bait pairings as non-binders on the basis that no binding between the respective nanobody and antigen has been reported; this status was not verified experimentally, so weak or cross-reactive binding cannot be fully excluded. We used this dataset to evaluate how consistently the studied confidence measures rank a true binding complex above bait complexes.

Detecting OOD sequences. To assess whether a confidence measure is sensitive to implausible, OOD sequences, we created an artificial nonsense variant of each of the 28 nanobodies by replacing the entire CDRs with alanine, using ANARCI for nanobody numbering (Dunbar and Deane, 2016) and IMGT CDR definitions (Lefranc et al., 2003). These OOD variants form a dedicated set that is evaluated independently from the true-binder and bait complexes. In total, depending on the nanobody, between 21% and 31% (median 27%) of the entire sequence was mutated to alanine residues. It has been found that extensive alanine repeats lead to higher misfolding and aggregation (Hernandez and Facelli, 2022; Darling and Uversky, 2017). We thus assume this extreme substitution to be both structure- and function-disrupting, and expect that these variants will not be suited for binding any of the 17 antigens in our dataset. An ideal confidence measure would assign an unfavourable score to such sequences. In contrast to scrambled CDRs, this extreme alanine substitution as a clear-cut OOD case has a well-defined expectation of the effect on folding and binding. Consequently, we treat the absence of a response to the alanine substitution in the confidence measures or structural prediction as one indicator of an implausibly handled sequence.

Alanine mutation sensitivity analysis. For a finer-grained analysis of mutation sensitivity, we devised an alanine scanning-inspired study. Alanine scanning is an established technique for identifying residues that contribute to protein function, stability, and shape (Morrison and Weiss, 2001). In nanobodies, the third heavy-chain CDR (CDR3) contributes most of the antigen-contacting amino acids (AAs) (Norman et al., 2020) and exerts the greatest influence on binding (Xu and Davis, 2000). We thus limit the scanning to this most influential region, which lets us sample more mutation configurations and obtain a more precise analysis than spreading the same number of mutations across all three CDRs. We therefore selected 13 nanobody–antigen complexes containing the shortest (PDB ID 9LAD, CDR3 length 9), median (PDB ID 8B1K, CDR3 length 17), and longest (PDB ID 9YC3, CDR3 length 22) CDR3s in our dataset. For each complex, we progressively mutated the CDR3 to alanine: for each number of mutations from 1 to the CDR3 length, we sampled 5 random mutation configurations. For 9LAD, 8B1K, and 9YC3, this yields 42, 82, and 107 configurations respectively, equally distributed along each CDR3. This setup allows us to analyse how each confidence measure responds to an increasing degree of mutational disruption.

Sequences from a camelid immunisation campaign against CD33. To complement the synthetic and structure-derived datasets, we incorporated a set of nanobody sequences obtained from a real-world camelid immunisation campaign using virus-like particles presenting full-length human CD33 (preclinics certified products GmbH), a clinically relevant antigen associated with acute myeloid leukaemia. All animal procedures were conducted in accordance with applicable national laws and regulations for animal welfare and were approved by the relevant local authorities. This dataset comprises 72,314 sequencing reads, from which 8,188 unique nanobody sequences were identified. To reduce redundancy while preserving sequence diversity, sequences were clustered at 95% sequence identity with at least 80% of the sequence length covered in the alignment, and the 100 most frequent clusters, comprising 4,366 unique sequences, were selected as representative candidates. In addition, five sequences obtained after multiple rounds of experimental enrichment were included, reflecting clones with increased binding affinity and selective pressure toward the target antigen. While the experimental enrichment drew on the full repertoire, the *in silico* screening was restricted to the 100 cluster representatives together with these five enriched sequences. As clustering was performed on the full sequence, while adequately preserving diversity in the test set, the CDRs within a cluster do not necessarily share 95% identity.

For the purpose of evaluation, we treat the representative cluster sequences as putative non-binders, as they have not undergone explicit enrichment for target binding, while the sequences obtained after the enrichment are considered real binders due to experimental selection pressure favouring CD33 affinity. As this label reflects the absence of enrichment rather than a direct binding measurement, the negative set may contain undetected weak binders.

This dataset is distinct from the others in that it originates from an *in vivo* affinity maturation process rather than the structural database. As such, it captures realistic sequence variability, experimental noise, and selection dynamics inherent to antibody discovery pipelines. The contrast between presumed non-binders and experimentally enriched binders provides a practically grounded test of whether scoring methods can predict sequence enrichment results and, as such, present a time and cost-reducing alternative.

### 2.2 Structure prediction models

We selected a representative subset of contemporary structure prediction models that have demonstrated strong performance in recent benchmarks (Xu et al., 2025; Chai Discovery et al., 2024; Passaro et al., 2025; Abramson et al., 2024; Teufel et al., 2023). The prediction settings and hyperparameters used for all models are presented in the implementation details of the Supplementary Material.

AF3 (Abramson et al., 2024) is a diffusion-based complex prediction model extending AF2 to jointly predict structures of biomolecular complexes, including proteins, nucleic acids, small molecules, ions, and modified residues in a single deep-learning framework. It exhibits reduced reliance on MSAs compared to AF2, while still benefiting from them, and achieves higher accuracy than specialised prior tools across protein–ligand, protein–nucleic acid, and antibody–antigen tasks.

Boltz-2 (Passaro et al., 2025) is a structural biology foundation model for biomolecular complexes targeting both structure prediction and binding affinity estimation, though affinity prediction is currently limited to small molecule–protein interactions. The model supports controllability features including experimental conditioning, inter-atomic distance constraints, and multi-chain template usage. Boltz-2 improves over Boltz-1 in structure prediction accuracy but remains slightly below AF3. Importantly, it is released with open-source code and open weights under a permissive license.

Chai-1 (Chai Discovery et al., 2024) is a foundation model for biomolecular structure prediction applicable to both AA sequences and SMILES strings. Like Boltz-2, it supports user-defined contact points and distance constraints to guide the generative process. Chai-1 supports both MSA-backed and single-sequence inference via protein language model embeddings, with MSA inclusion generally improving prediction quality. As for Boltz-2, this model is available with both open-source code and open weights. Note that this is not yet the case for its successor Chai-2 (Chai Discovery et al., 2025).

IntFold (The IntFold Team et al., 2025) is a controllable foundation model whose lightweight, modular design supports both general and specialised biomolecular structure prediction. It achieves accuracy comparable to AF3 on diverse biomolecules and enables controllability via user-defined constraints. Through *post hoc* fine-tuning, it outperforms Boltz-2 on protein–ligand binding affinity estimation. Again, this model is available with open-source code and open weights.

Models not included. We did not include purely language-model-based methods such as OmegaFold (Wu et al., 2022) and ESMFold (Lin et al., 2023), nor the MSA-based models RoseTTAFold (Baek et al., 2021), RoseTTAFoldNA (Baek et al., 2024), Protenix (Protenix Team et al., 2026), or HelixFold 3 (Liu et al., 2024), as these have been shown to be outperformed by Chai-1, Boltz-2, or AF3 with respect to structure prediction quality (Chai Discovery et al., 2024; Hýsková et al., 2026; Xu et al., 2025). The MiniFold (Wohlwend et al., 2025) model was considered in a preliminary study, but its restriction to single-chain predictions makes it unsuitable for our analysis. Finally, we did not include SimpleFold (Wang et al., 2025), SeedFold (Zhou et al., 2025), or Chai-2 (Chai Discovery et al., 2025), all of which were released concurrently with this study. We also excluded antibody-specific structure predictors such as AbLooper (Abanades et al., 2022a) and IgFold (Ruffolo et al., 2022). These predict only the antibody Fv structure rather than the nanobody–antigen complex (AbLooper models only the CDR loops rather than the full Fv), and they do not provide binding-oriented confidence measures, placing them outside the scope of our complex-scoring analysis. The AbLooper successor ImmuneBuilder2 (ABodyBuilder2) (Abanades et al., 2022b) matches AlphaFold-Multimer (Evans et al., 2022) on antibody structure prediction, which AF3 in turn substantially outperforms on antibody–antigen prediction (Abramson et al., 2024).

### 2.3 Confidence measures

The quality of a structure prediction cannot be assessed directly without reference to experimental data. A range of surrogate metrics has therefore been developed to measure model confidence and structural plausibility. Such confidence measures quantify the internal confidence of a model in its prediction or its predicted agreement with known structural or biophysical properties. Prior studies have demonstrated that these confidence measures can additionally be employed to predict whether a candidate binds the target of interest (Bennett et al., 2023; Vázquez Torres et al., 2025; Teufel et al., 2023). Here, we present measures extracted directly from the evaluated structure prediction models, together with additional measures derived from aggregations of these predictions.

The pLDDT (Abramson et al., 2024) is a local, per-atom confidence measure that estimates how well a predicted structure would match an experimentally resolved structure. It is local in the sense that only atom pairs within a fixed distance of the current atom are considered (Abramson et al., 2024), and it is not sensitive to global alignment, which can be a limitation for estimating overall packing (Jumper et al., 2021). pLDDT is reported on a scale of 0–100, where higher values indicate greater confidence.

The pAE is a pairwise confidence measure that predicts the positional error of one residue when the structure is aligned with respect to the frame of another residue. It is therefore sensitive to global alignment and is particularly useful for analysing interactions within subsets of atoms, such as protein interfaces (Abramson et al., 2024). The predicted Distance Error (pDE) is analogous to the pAE but estimates pairwise absolute distances between atoms (Abramson et al., 2024). Lower pAE and pDE indicate higher confidence.

The predicted Template Modelling (pTM) score aggregates the pAE matrix into a single normalised whole-structure confidence scalar (Jumper et al., 2021). Building on pAE, it is more sensitive to global topology than to local changes. Note that in protein complexes, the pTM can be dominated by the larger chain, or parts of the complex that are not relevant for binding (Dunbrack, 2025). Higher pTM indicates higher confidence.

Rosetta’s ΔG (dG) separated is obtained by applying Rosetta’s InterfaceAnalyzer to a predicted complex. It approximates the change in binding free energy in Rosetta Energy Units when separating the interface-forming chains. Note that predicted complexes are preprocessed using Rosetta’s FastRelax functionality to remove clashes. Lower ΔG -separated values indicate stronger predicted binding affinity. In contrast to other measures presented here, ΔG is not directly a confidence measure predicted by a neural network. Instead, it is derived based on the predicted complex structure and uses energy terms derived from physics force fields to give an estimate of binding quality. For simplicity of notation in this work, when we refer to confidence measures, this includes ΔG measures.

#### 2.3.1 Interface-specific confidence measures

Interface-specific variants have been proposed for many of the above measures to focus attention on the protein–protein interaction site. Importantly, not all interface measures are defined identically. The interface predicted Template Modelling (ipTM) by Evans et al. (2022) defines the interface as any two residues from different chains. The interface predicted Alignment Error (ipAE) by Teufel et al. (2023) additionally requires a distance cutoff of 3.5 Å. Boltz-2 includes interface variants like the interface predicted Distance Error (ipDE), interface predicted Local Distance Difference Test (ipLDDT), and ipTM in which only atom pairs from different chains and below a distance cutoff are considered, though the technical report does not specify the exact cutoff value. For Rosetta’s InterfaceAnalyzer, the interface distance defaults to 10 Å. More broadly, interface-distance definitions vary widely across the literature, ranging from 4 Å in DiscoTope-3.0 (Høie et al., 2024) and Yin and Pierce (2024), and 5 Å in DockQ (Basu and Wallner, 2016) to the 10 Å Rosetta default (Alford et al., 2017), and differ further in whether the distance is measured between any atom pair or only between C α atoms.

Many structure prediction models return only final aggregated values for confidence measures such as pLDDT or pTM, making source code modifications necessary to extract interface-specific variants.

For our analysis, we computed two sets of custom interface measures. First, we introduce interacting interface versions of pAE and pDE, defined analogously to those of Teufel et al. (2023) and Passaro et al. (2025) but using Rosetta’s 10 Å distance cutoff. We measure this cutoff between C α atoms and deliberately adopt the conservative 10 Å Rosetta default rather than the 3.5 Å cutoff used by Teufel et al. (2023): the latter is presumably an all-atom cutoff and would be unsuitable for a C α -based definition, since even consecutive backbone C α atoms lie about 3.8 Å apart (Chakraborty et al., 2013). A cutoff that is too tight can moreover leave an interface undetected entirely, particularly for predictions in which the chains are docked only loosely. Note that the optimal cut-off value is likely to depend on the model, the measure and the antigen, and should be adapted by practitioners to fit their respective use cases. Second, we introduce CDR versions of pAE, pDE, and pLDDT, which restrict attention to residue pairs $a_{i,j}$ where both i and j lie within the CDRs as defined by IMGT (Lefranc et al., 2003). These measures put focus exclusively on the regions typically targeted by generative design methods (Cowen-Rivers et al., 2022; Khan et al., 2023; Liu et al., 2020; Luo et al., 2022; Zhou et al., 2024). For the pLDDT we sum over all residues within the CDRs. By disregarding antigen chain residues entirely, the CDR measures assess the plausibility of the CDRs in isolation.

#### 2.3.2 Combined and derived measures

When using ipTM, disordered regions not involved in the interface can substantially lower the overall score. To address this, Dunbrack (2025) proposed an adaptation of the pTM score, the interface predicted TM Score based on Aligned Errors (ipSAE), that considers only residue pairs whose pAE falls below a user-chosen threshold, thereby excluding low-confidence residues from the calculation. Following the same gist, the Local Interaction Score (LIS) (Kim et al., 2024) focuses on areas with a pAE below a fixed threshold. However, while LIS uses a linear kernel with a single hard threshold and averages across all inter-chain residue pairs, ipSAE is based on a non-linear pTM kernel and per-residue averaging, making it more sensitive to interface geometry.

Bryant et al. (2022) proposed pDockQ, which estimates DockQ scores, a continuous quality score for protein–protein docking models (Basu and Wallner, 2016), from AF2-derived measures. Using pDockQ, the authors were able to distinguish 1,481 truly interacting from 3,989 non-interacting protein pairs with high ROC AUC. Zhu et al. (2023) subsequently proposed pDockQ2, which incorporates pAE alongside pLDDT to produce a per-chain interface quality estimate for multimeric complexes.

Each evaluated model additionally computes an internal aggregate score used to rank replicates. For Boltz-2, this confidence score combines pLDDT and ipTM (or pTM for single-chain predictions). For AF3 and IntFold, the ranking score incorporates ipTM, pTM, a disorder estimate, and a clash indicator (Abramson et al., 2024). Similarly, for Chai-1, the aggregate score combines pTM, ipTM, and an estimate of inter-chain clashes. For notational simplicity, we will refer to these aggregate scores as *aggr. Score* for any model.

#### 2.3.3 Comparing confidence measures

The analysed confidence measures operate on different scales and have opposing optimisation directions (minimisation vs. maximisation). To enable statistically valid comparison, we used the Mann–Whitney U test to determine, for each antigen, the probability that a given confidence measure ranks a true binding complex above a bait complex or a realistic complex above an OOD complex. Specifically, we computed the ROC AUC as a normalised U-statistic to account for varying numbers of valid samples per antigen. We adopt the ROC AUC as our primary metric because it matches the screening objective of this work: reducing a large candidate pool to a smaller set enriched for true binders, while only a budget-limited number of top candidates can be advanced to costly experimental validation. In this setting, the number of true binders, and hence the baseline binding probability, is unknown and varies between antigens. As the probability that a true binder is ranked above a non-binder, the ROC AUC is invariant to this baseline probability and quantifies a measure’s general ability to discriminate binders from non-binders, rather than the prevalence-specific precision among the top-ranked candidates. For practitioners instead interested in measures that reliably place true binders at the very top of the ranking, and that are sensitive to the size of the true-binder set, the Precision–Recall Area Under the Curve (PR-AUC) is better suited. We report it as a complementary metric in the appendix. For each pair of confidence measures, we then tested whether one achieves a significantly higher ROC AUC across antigens using a paired Wilcoxon signed-rank test. Such statistical testing was absent from prior analyses (Teufel et al., 2023; Bryant et al., 2022).

## 3 Results

We organise the results into five parts: true binder identification, OOD detection, an alanine mutation sensitivity analysis, and an overview of measurements that are suited to simultaneously detect true binders and OOD sequences. Lastly, we present results on sequences obtained from the *in vivo* immunisation campaign against CD33.

### 3.1 Identifying true binders

To evaluate if confidence measures derived from structure prediction models are suited for ranking true binders above non-binders, we predicted structures and corresponding confidence measures of 28 true complexes and 482 non-binding complexes for each of the four analysed structure prediction models, with five replicates each. We then computed the ROC AUC per confidence measure, giving the probability that a random true binding nanobody is ranked above a randomly chosen non-binding nanobody given an antigen.

**FIGURE 2:**
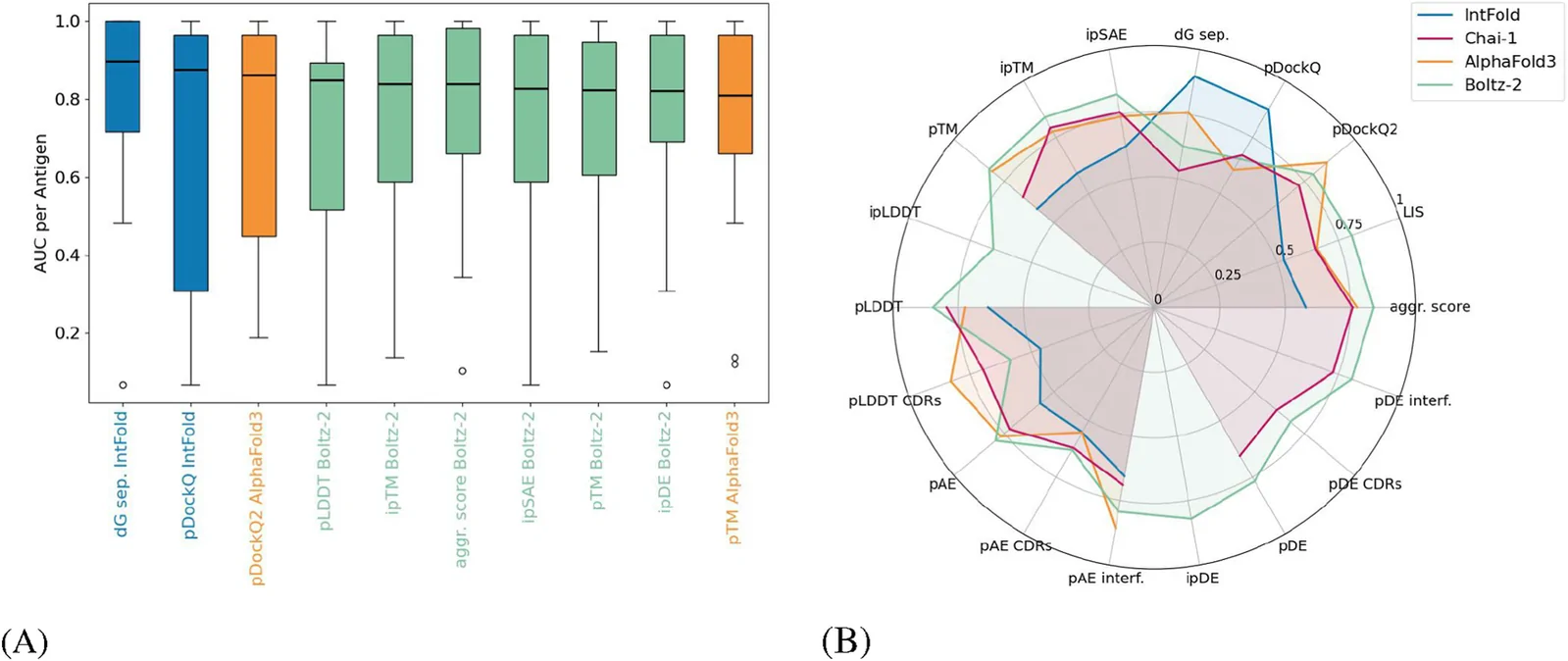
ROC AUC scores for 60 confidence measures derived from AF3, IntFold, Boltz-2, and Chai-1. ROC AUC gives the probability that a random true binding nanobody is ranked above a randomly chosen non-binding nanobody given an antigen. We compute ROC AUC distributions over 17 antigens with a total of 28 true binding complexes and 482 non-binding bait complexes. **(A)** Top 10 confidence measures by median ROC AUC. For this comparison, we excluded our novel CDR and interacting interface specific versions of confidence measures, to facilitate a comparison of confidence measures directly returned by the respective models. See Supplementary Figure S1 and Supplementary Figure S2 for full details. **(B)** Median ROC AUC of all analysed confidence measures, facilitating a direct comparison of structure prediction models per confidence measure of interest. Here we included our 4 new CDR and interacting interface measures per structure prediction model.

Figure 2 shows the distribution of ROC AUC over all 17 antigens. In Figure 2A, we observe that the top 10 confidence measures by median ROC AUC include measures from all four models, with Boltz-2 contributing the largest share. The three single highest-ranked measures, however, are not derived from Boltz-2: IntFold’s physics-based Rosetta ΔG ranks first overall (median ROC AUC 0.90), followed by pDockQ from IntFold (0.88) and pDockQ2 from AF3 (0.86). The strong performance of pDockQ and pDockQ2 is consistent with their design purpose, as both estimate the DockQ score (Basu and Wallner, 2016), a measure of the quality of a predicted protein–protein binding conformation (Bryant et al., 2022; Zhu et al., 2023). IntFold’s ΔG , the only measure based on physical interactions rather than a neural confidence estimate, ranks first even though IntFold’s neural confidence measures otherwise tend to lag. Globally oriented confidence measures that capture long-range structural dependencies, such as pAE, pDE, and scores derived from these, including pTM and pDockQ, generally perform better than local measures, such as pLDDT. For a presentation of all 60 analysed confidence measures, see Supplementary Figure S1.

Coinciding with the top 10 confidence measures, Figure 2B shows that for confidence measures shared by all analysed structure prediction models, Boltz-2 achieves the highest ROC AUC for many of these shared measures. AF3 and Chai-1 perform well in different measures, while IntFold’s neural confidence measures tend to lag behind, with the notable exception of its Rosetta ΔG and pDockQ discussed above. Interface-specific variants, including ipDE, ipTM, and our interacting interface versions of pAE and pDE, indicated by the interf. Measure naming, also perform competitively. That such global and interface-oriented measures rank true binders well is consistent with Abramson et al. (2024), who report that AF3’s ipTM correlates with interface accuracy as measured by DockQ. In contrast, all CDR versions of pAE, pDE, and pLDDT, indicated by CDRs measure naming, perform worse than their unrestricted counterparts.

As our benchmark is class-imbalanced, we additionally report the PR-AUC as the complementary, prevalence-dependent metric motivated in Section 2.3.3. The ranking of confidence measures changes moderately under PR-AUC (Spearman ρ=0.66 between the per-metric median ROC AUC and median PR-AUC across antigens), with AF3-derived measures such as pLDDT and pTM rising to the top (Supplementary Figure S8; Supplementary Figure S9).

Note that while differences in median ROC AUC are visible descriptively in Figure 2B, these differences are statistically significant in only a few pairwise comparisons, see Supplementary Figure S2 for a significance analysis.

**FIGURE 3:**
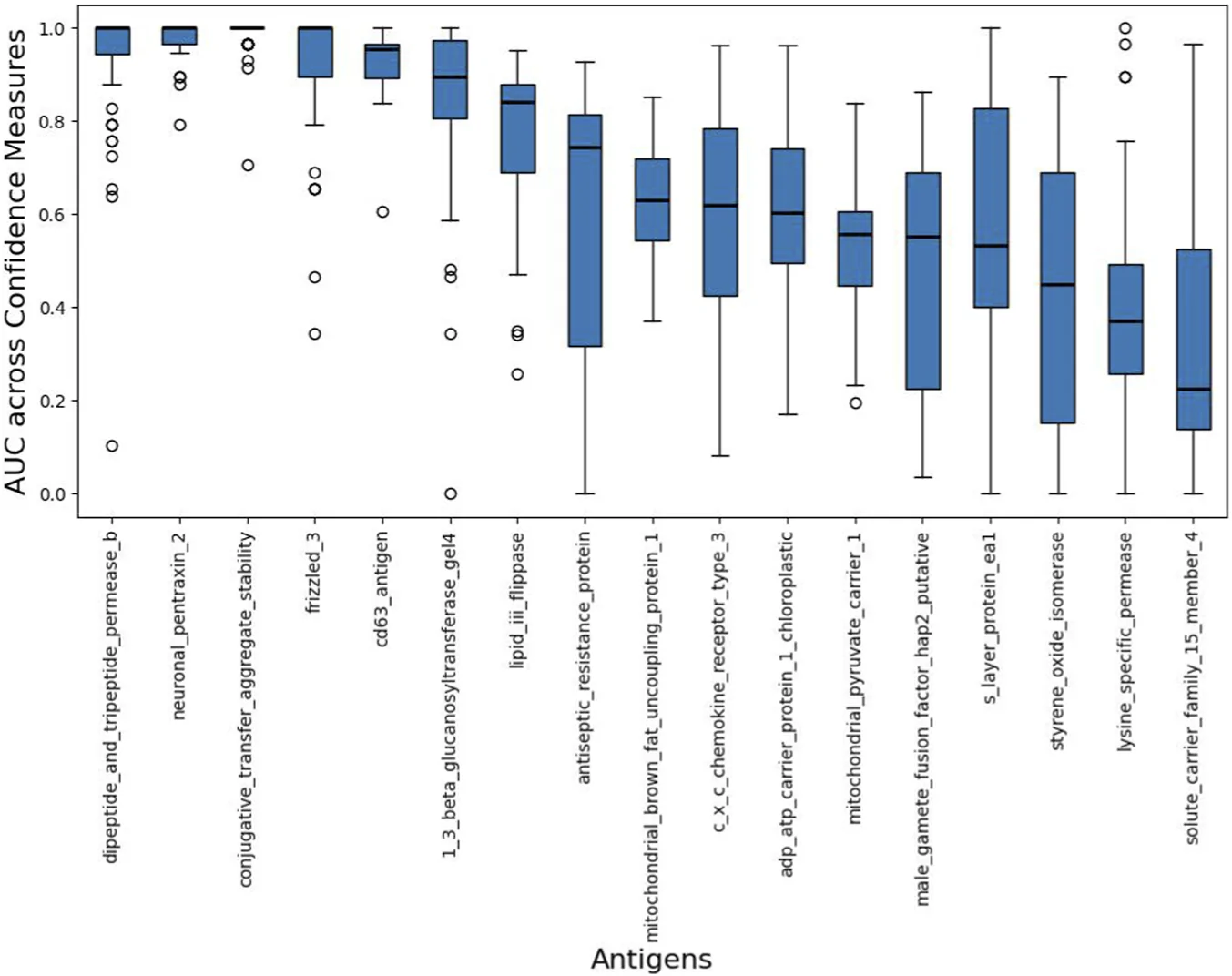
Boxplot of ROC AUC over all confidence measures, excluding our CDR and interacting interface specific version, per antigen when separating true binders from bait sequences.

This insignificance can in part be attributed to the large variation in ROC AUC across antigens, illustrated in Figure 3. For some antigens, almost all confidence measures achieve high ROC AUC, very consistently ranking the true binders above non-binding complexes. However, for many antigens, most confidence measures achieve a low ROC AUC, pointing to a generalisation limitation of current structure prediction models. To investigate the source of this variation, we analysed the correlation between median ROC AUC and both antigen length and nanobody length; neither was significant. We further explored whether antigen sequence similarity to structures deposited in the RCSB before 1 June 2023, could explain the ease of prediction, but found no clear pattern. We did, however, find that an antigen’s intrinsic predictability is reflected in the models’ own confidence when folding the antigen alone. Across all structure prediction models, the median ROC AUC achieved per antigen correlates with the structural confidence of the antigen-only prediction: higher antigen-only pLDDT and lower antigen-only pAE are associated with better true binder identification. The pAE correlation is significant for every model (Spearman ρ between −0.51 and −0.63 , all p<0.05 , n=17 ), while the pLDDT correlation shows a consistent positive trend ( ρ up to 0.49). The relationship persists under a leave-one-model-out control that excludes each model’s own complex-mode measures from the median ROC AUC, indicating that antigen-only structural confidence is a simple, largely model-agnostic indicator of whether an antigen is amenable to structure-based *in silico* screening. Identifying a reliable way of deciding whether an antigen is suited for analysis by structure prediction models is thus interesting for future research.

**FIGURE 4:**
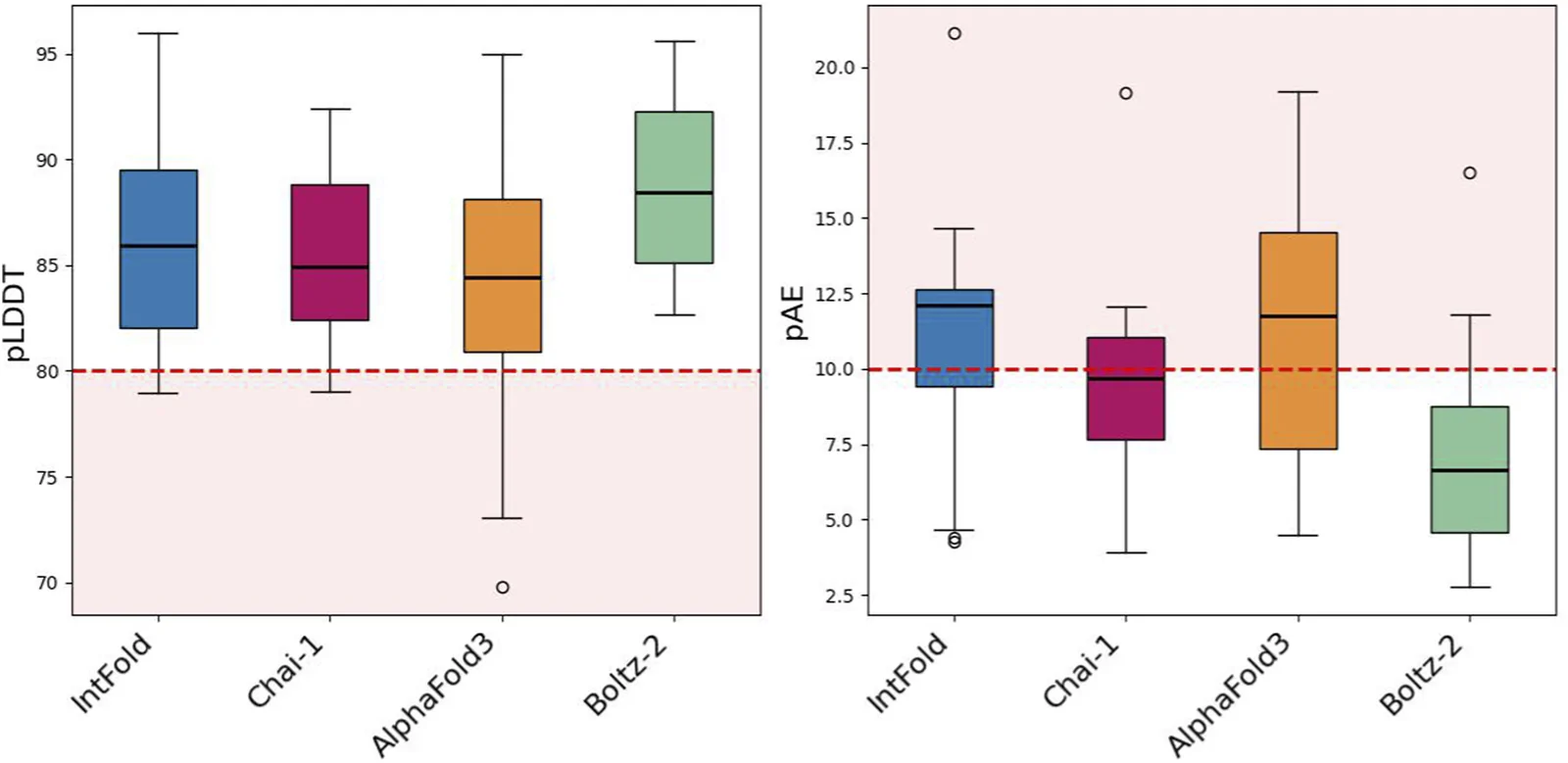
Distribution of pLDDT and pAE for all analysed models. We observe that up to 25% of true binders would have been discarded using the pLDDT threshold and up to 75% using the pAE threshold proposed in literature. The red bar marks the thresholds.

In recent publications, using confidence measures for candidate filtering, hard thresholds on confidence measures were proposed to discard candidates. Vázquez Torres et al. (2025) filtered using both a threshold of >80 for pLDDT and <10 for pAE before screening candidates in wet-lab experiments. Watson et al. (2023) used a threshold of >80 for pLDDT to define *in silico* experiment success. Given the strong fluctuation over antigens, we analysed the distribution of pLDDT and pAE as displayed in Figure 4. We found that up to 25% of true binders would have been discarded using the pLDDT threshold and up to 75% using the pAE threshold proposed in literature. This indicates that variable thresholds or model and antigen-specific thresholds are beneficial.

### 3.2 Identifying Out-Of-Distribution sequences

To evaluate if confidence measures can identify and thus discriminate against extreme OOD sequences, we assembled a second dataset, separate from that of Section 3.1: for each of the 28 nanobodies, the entire CDRs (all three) were replaced by an equal number of alanine residues, yielding 28 OOD complexes. For each antigen, all nanobodies (binding any antigen) are then compared against the all-alanine variant. These extreme mutations tend to lead to misfolding structures and aggregation (Hernandez and Facelli, 2022; Darling and Uversky, 2017). We then computed, for each antigen, the ROC AUC representing the probability of ranking any random nanobody among the 28 real nanobody sequences above the OOD sequence.

**FIGURE 5:**
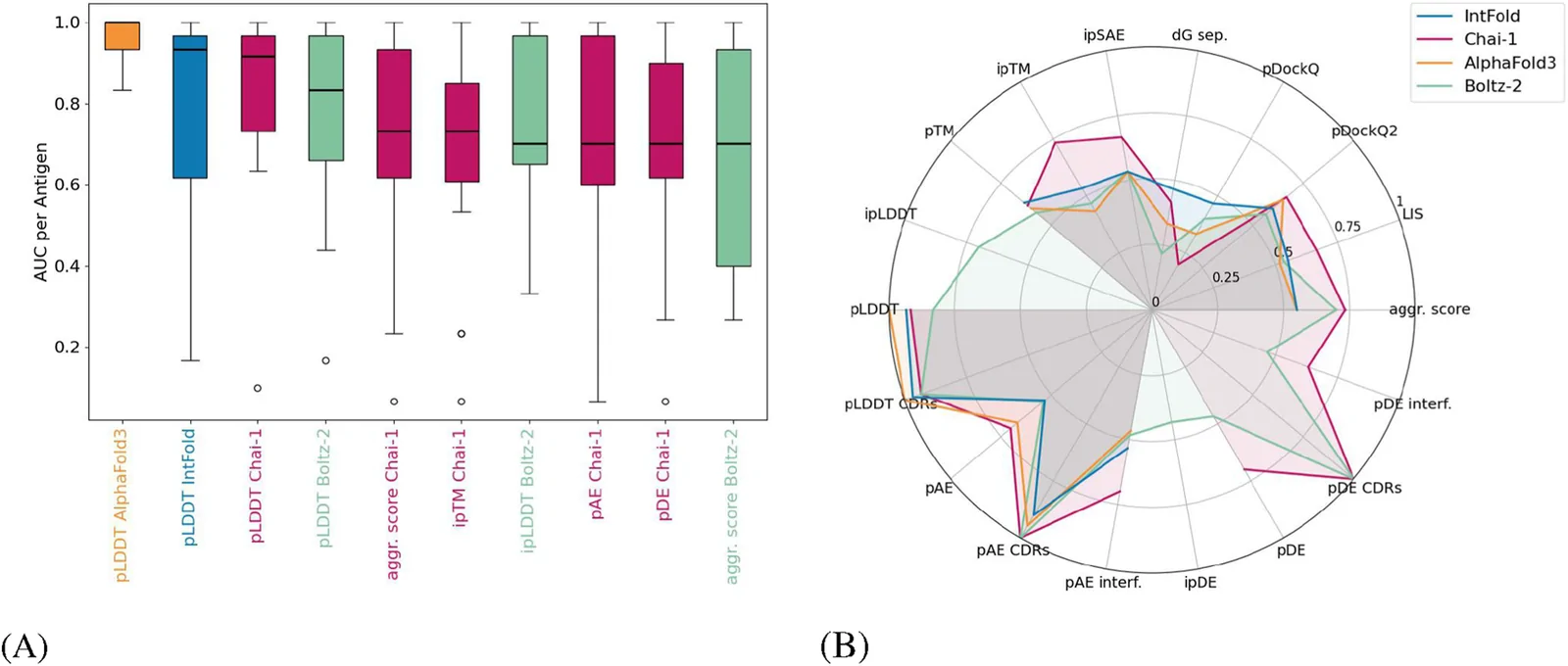
ROC AUC scores for 60 confidence measures derived from AF3, IntFold, Boltz-2, and Chai-1. ROC AUC gives the probability that a random real nanobody is ranked above a OOD nanobody, where the entire CDR3 consists of alanine, given an antigen. We compute ROC AUC distributions over 17 antigens with a total of 28 real nanobodies and a single extreme OOD sequence per antigen. **(A)** Top 10 confidence measures by median ROC AUC. For this comparison, we excluded our novel CDR and interacting interface specific versions of confidence measures, to facilitate a comparison of confidence measures directly returned by the respective models. See Supplementary Figure S3 and Supplementary Figure S4 for full details. **(B)** Median ROC AUC of all analysed confidence measures, facilitating a direct comparison of structure prediction models per confidence measure of interest. Here we included our 4 new CDR and interacting interface measures per structure prediction model.

As seen in Figure 5A, out of the confidence measures predicted by the model, the pLDDT, focusing on local structure, consistently achieves the highest ROC AUC, with scores above 0.83. This is consistent with the observation by Abramson et al. (2024) that AF3 can erroneously predict ordered structure for disordered regions, but typically assigns such hallucinated regions very low confidence. Local measures such as pLDDT thus flag the implausible all-alanine CDRs even when the overall fold remains superficially plausible. In contrast to Section 3.1, we observe a decreased performance of globally orientated confidence measures and an increase in performance for measures predicted by Chai-1. The physics-based ΔG measures, in particular, are among the lowest-ranked for OOD detection across all models (median ROC AUC between 0.22 and 0.47), as a relaxed all-alanine complex can still yield a favourable interface energy. This performance in OOD detection can further be improved by using CDR specific versions of pAE, pDE, and pLDDT. In our experiments, such versions achieve a median ROC AUC of up to 1.0 as visualised in Figure 5B. Note that this almost perfect separation represents an upper bound and relies on the prior knowledge that changes occur in the CDR.

In contrast to Section 3.1, the differences are often statistically significant. The pLDDT predicted by AF3 achieves a significantly higher ROC AUC across the 17 antigens than any other confidence measure. Further, pLDDT scores by any structure prediction model often achieve a significantly higher ROC AUC than other confidence measures. When also including the CDR specific measures, several measures reach a median ROC AUC of 1.0. This perfect-separation upper bound is attained by the CDR pDE and pAE of Chai-1 and Boltz-2 and the CDR pLDDT of AF3, matching the model-returned pLDDT of AF3, with none significantly outperforming the others. For full details of the significance analysis and the performance of all 60 evaluated measures, we refer the reader to Supplementary Figure S3 and Supplementary Figure S4.

Accounting for the class imbalance, we additionally report the PR-AUC. The ranking of confidence measures is largely preserved under PR-AUC (Spearman ρ=0.86 between the per-metric median ROC AUC and median PR-AUC across antigens) (Supplementary Figure S10; Supplementary Figure S11).

### 3.3 Computational alanine scanning

In Section 3.2, we only considered a single extreme OOD sequence per nanobody. This does, however, not capture the sensitivity of the analysed confidence measures with respect to gradually increasing mutational disruptions. Inspired by alanine scanning, suited for identifying residues that contribute to protein function, stability, and shape, we progressively mutated the residues in nanobody CDR3s to alanine and observed the effects on the outputs of protein structure-prediction models.

**FIGURE 6:**
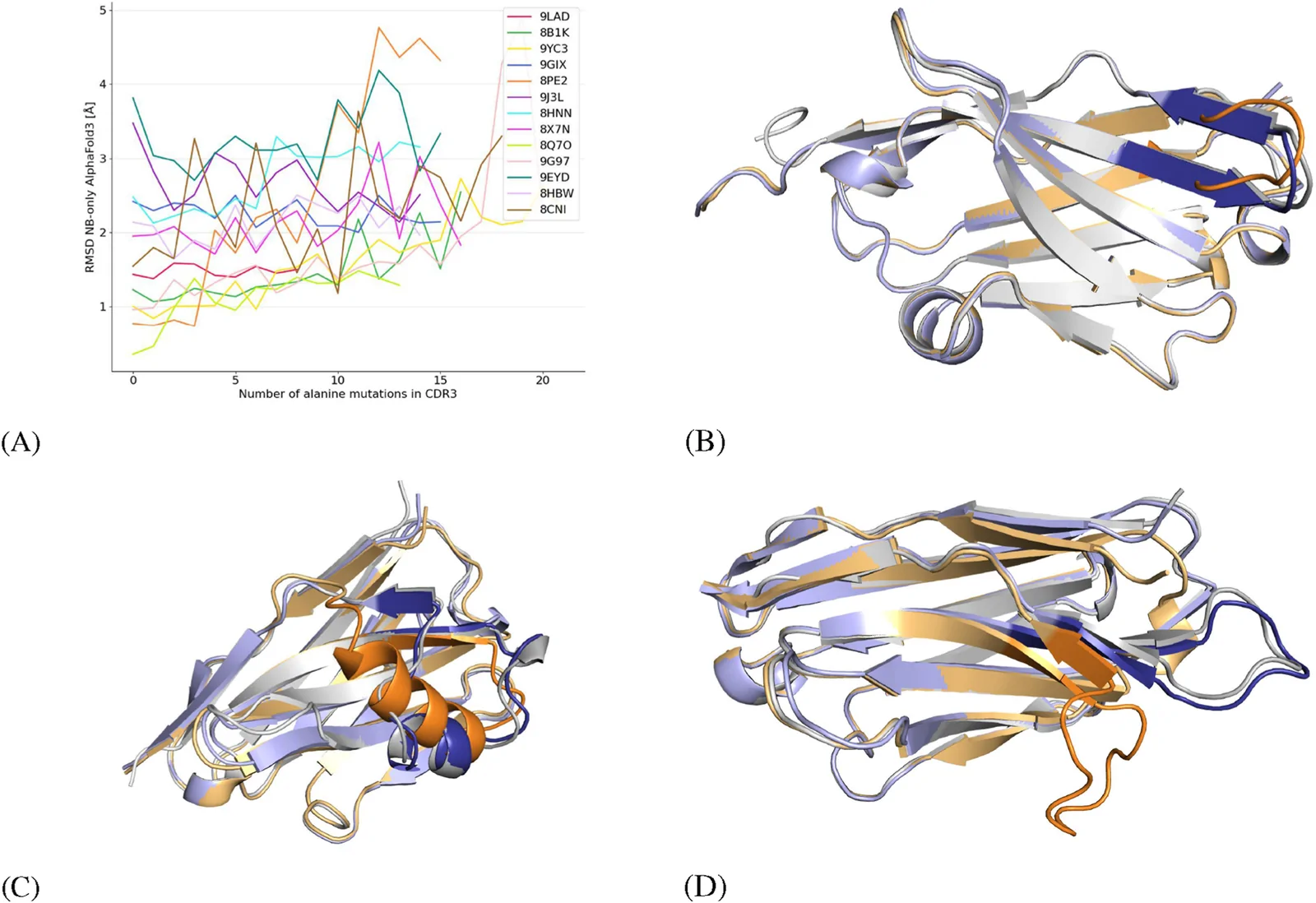
**(A)** RMSD between the original nanobodies and nanobodies with an increasing number of alanine mutations in the CDR3, predicted by AF3. **(B–D)** Original nanobody from 9LAD, 9YC3, and 8PE2 (grey), predicted nanobody using AF3 (blue), and predicted nanobody when mutating all CDR3 residues to alanine (orange). CDR3 residues are highlighted in a darker colour.

We start by analysing whether a change in the CDR3 residues induces a change in the predicted structure. Exemplarily, we present results for structures predicted using AF3. Here we report the change in the predicted nanobody structure; the complementary change in the predicted *antigen* structure, measured against the experimentally determined antigen, is presented in Supplementary Figure S6. In Figure 6A, we can observe that for many, though not all, antigens the RMSD between the original nanobody and the predicted nanobody structure does not consistently increase with the number of mutations, indicating that the structure prediction model does not always reflect the growing deviation from realistic sequences. Even heavily mutated CDR3s can result in predicted structures that remain close to the original conformation. For these complexes, the limited structural response is consistent with AF3’s reported tendency to hallucinate ordered, confident-looking structure for inputs it should treat as disordered (Abramson et al., 2024). Figures 6B–D illustrates changes in predicted structures for the shortest CDR3 (B), longest CDR3 (C), and the most pronounced structural deviation observed (D), with respectively 9, 22, and 15 mutated residues. We observe that while CDR3s are adapted, e.g., exhibiting a prolonged α -helical segment in Figure 6C, the nanobodies are still predicted to adopt a plausible fold. On the one hand, Bernacki and Murphy (2011) found that such prolonged α -helical segments occur for increased uninterrupted alanine repeats. On the other hand, such sequences also tend to be disordered and have increased aggregation propensities, indicating that the almost unchanged structures are unlikely (Bernacki and Murphy, 2011; Darling and Uversky, 2017; Hernandez and Facelli, 2022). Experimental validation would be required to determine whether such sequences are indeed structurally viable.

Note that for *de novo* design tasks, there are no known reference nanobody structures, and as such, the RMSD cannot be used. Therefore, confidence measures should capture uncertainty even without access to known structures.

**FIGURE 7:**
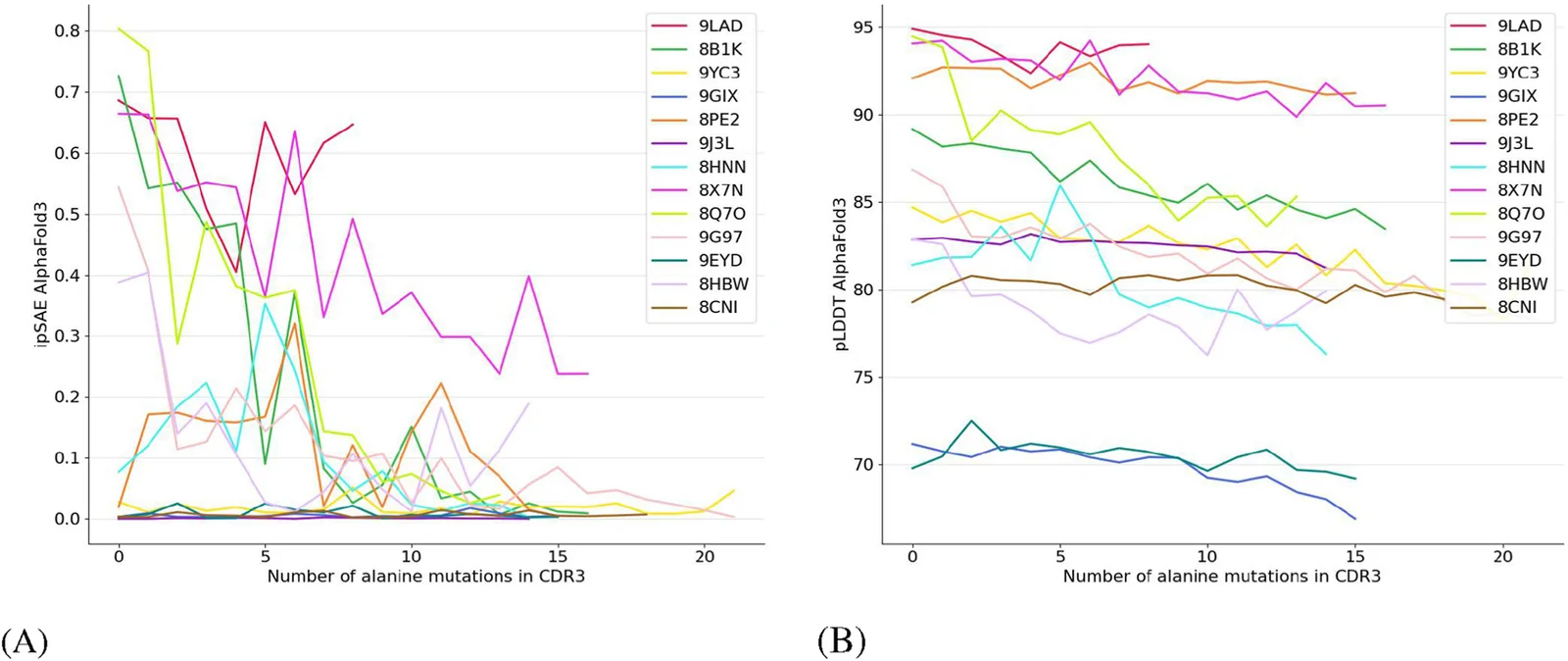
Confidence measure sensitivity to progressive CDR3 alanine mutation, both derived from AF3. **(A)** ipSAE of AF3 (the worst performing measure): counterintuitively increases with the number of mutations, indicating higher confidence as the binder is degenerating. **(B)** pLDDT of AF3: decreases with mutation count, behaving as desired.

Figure 7 illustrates the behaviour of two representative confidence measures of AF3 as the CDR3 is progressively mutated to alanine. ipSAE of AF3, the worst-performing confidence measure, exhibits an increasing confidence with an increased number of mutations. We attribute this to ipSAE only considering a subset of residue pairs with a pAE below a threshold (20 in our setting). Through progressive mutation, more and more CDR residue interactions will be ignored, leaving only high-confidence framework regions. This highlights a weakness of region-independent analysis. In contrast, the pLDDT of AF3, the strongest measure for OOD detection (Section 3.2), decreases as the CDR3 is mutated, correctly reflecting the growing implausibility of the sequence.

**FIGURE 8:**
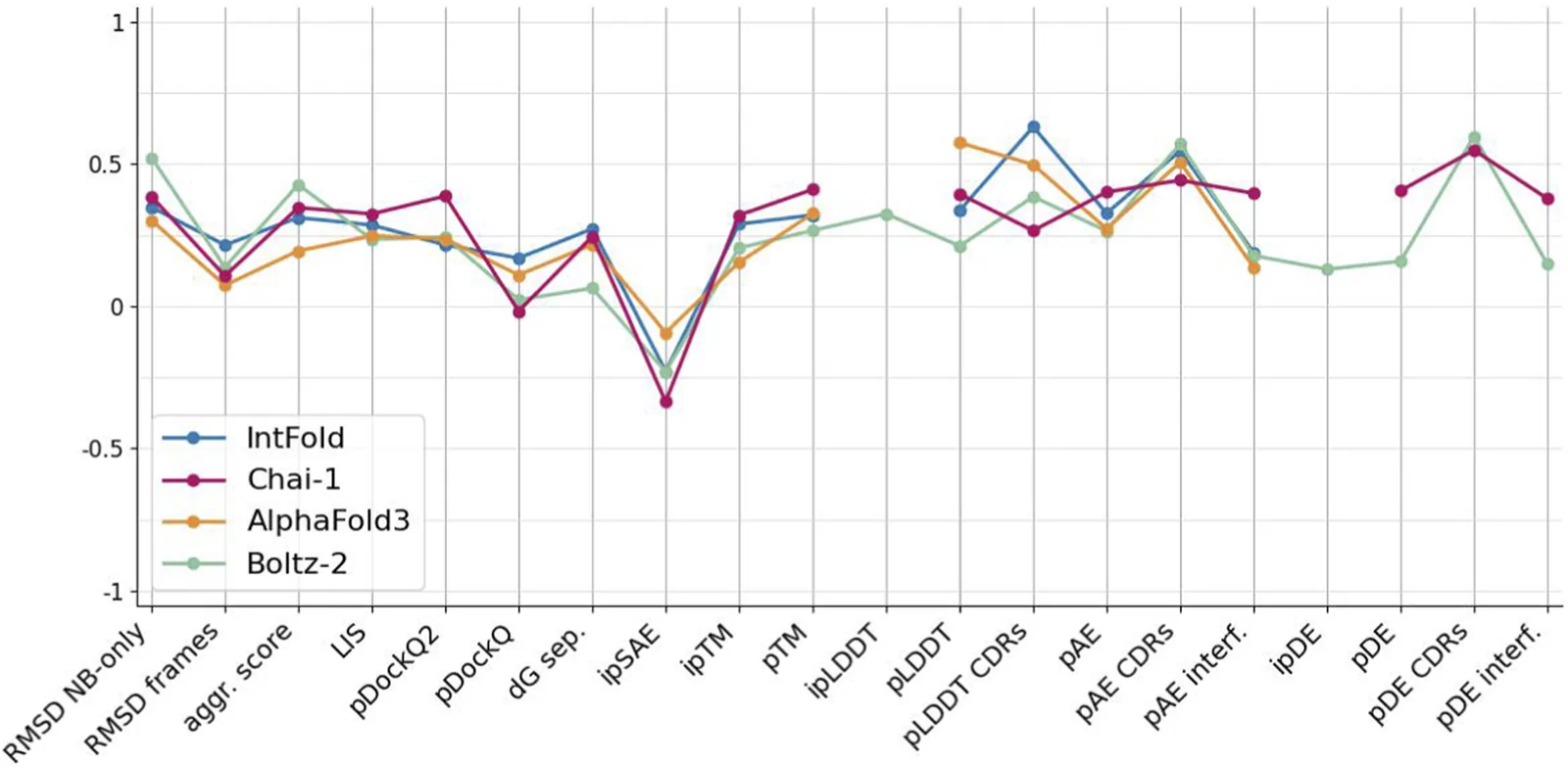
Distribution of Spearman correlations between the number of CDR3 mutations and confidence measure score, across all analysed antigens.

To summarise this analysis across all antigens and confidence measures, Figure 8 shows the median of Spearman correlations between the number of mutations and each confidence measure score. For an overview of the number of statistically significant correlations per measure, see Supplementary Figure S5. Correlations are low for the majority of measures, consistent with prior findings that AF2 does not capture the effect of missense mutations (Buel and Walters, 2022; Pak et al., 2021). Measures with higher correlations largely overlap with those that also achieve high ROC AUC in the OOD detection analysis (Section 3.2), indicating that the extreme OOD test is a suitable proxy.

### 3.4 Are confidence measures suited for both tasks?

**FIGURE 9:**
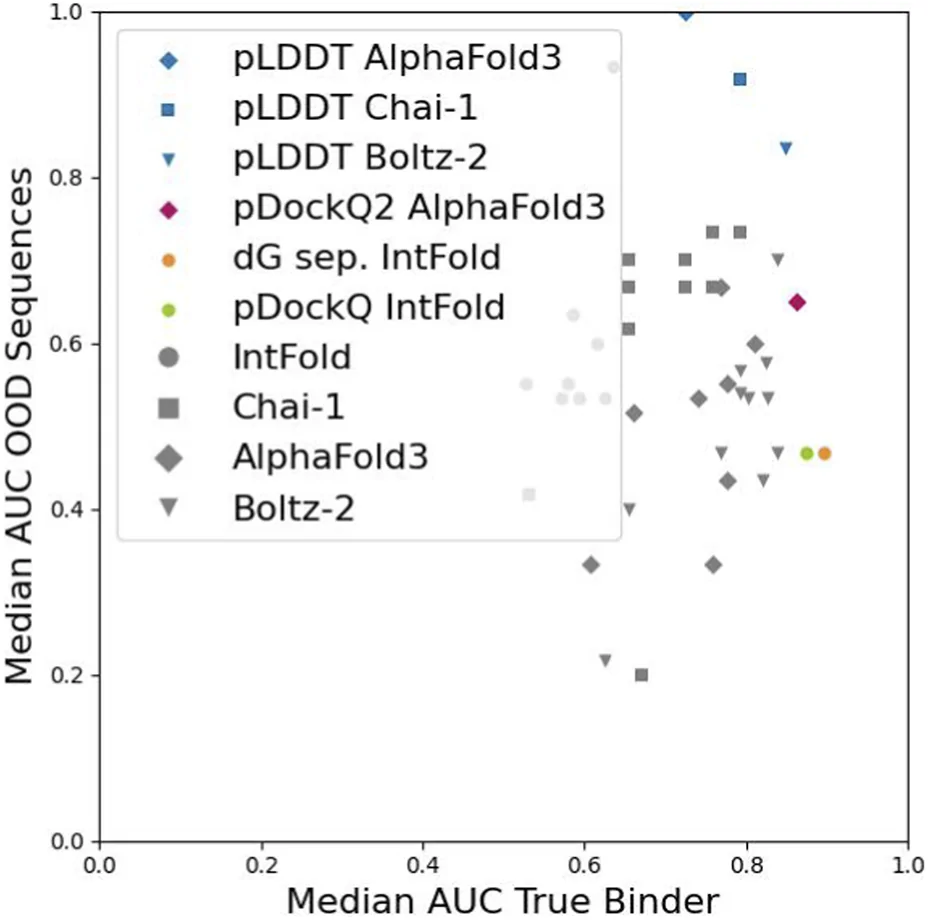
Scatter plot of median ROC AUC for true binder identification (x-axis) vs. OOD detection (y-axis) across all evaluated confidence measures.

Figure 9 presents the trade-off between true binder identification and OOD detection performance across all confidence measures. Performance measures are widely scattered, typically excelling at only one of the two tasks, while no single confidence measure achieves optimal performance on both simultaneously. Instead, a set of six Pareto-optimal confidence measures emerges. Among these, the pLDDT predicted by AF3, Chai-1 and Boltz-2 perform well with respect to OOD detection, whereas IntFold’s physics-based Rosetta ΔG and pDockQ, together with pDockQ2 from AF3, trade off this capability in favour of improved true binder identification.

The observed spread in Figure 9 is consistent with the findings from the previous sections. In Section 3.1, measures capturing global and interface-level consistency showed stronger performance, whereas in Section 3.2 and Section 3.3, measures sensitive to local structural confidence and perturbations were more effective. This divergence explains why measures that perform well in one setting often fail in the other, resulting in the absence of a single dominant measure across both tasks.

In practical settings, especially in screening or design pipelines, these findings suggest that relying on a single confidence measure is insufficient. The six Pareto-optimal measures identified in Figure 9 offer a practical starting point: practitioners focused on binding enrichment should favour IntFold’s Rosetta ΔG or pDockQ, or pDockQ2 from AF3, while those screening AI-generated sequences for structural plausibility should add pLDDT from AF3, Chai-1 or Boltz-2. Combining both objectives into a single weighted score and using measures outside the Pareto-front is feasible, but should include antigen-specific calibration, which remains an open challenge given the limited availability of ground-truth binding data.

### 3.5 Predicting nanobody enrichment in a camelid immunisation campaign against CD33

**FIGURE 10:**
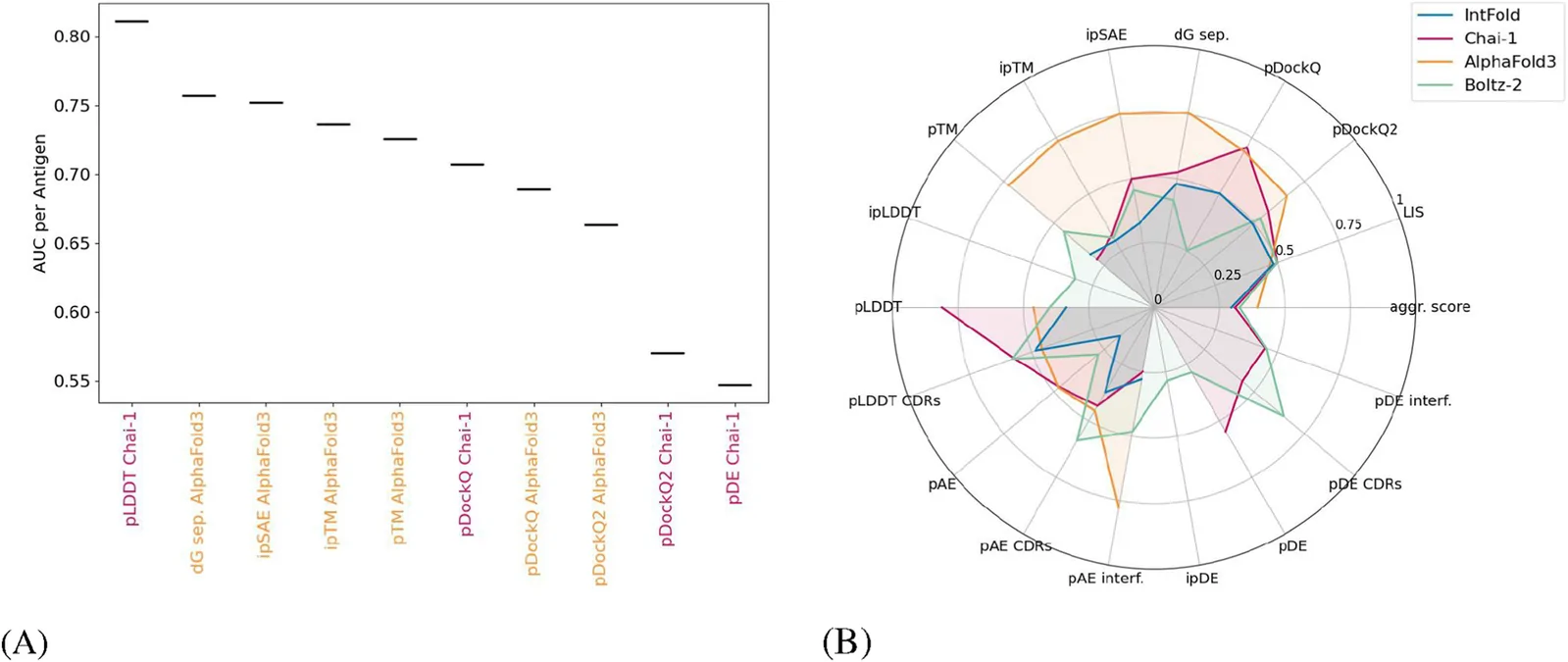
ROC AUC scores for 60 confidence measures derived from AF3, IntFold, Boltz-2, and Chai-1. ROC AUC gives the probability that a random nanobody confirmed to bind CD33 is ranked above a random nanobody contained in the campaign. **(A)** Top 10 confidence measures by median ROC AUC. For this comparison, we excluded our novel CDR and interacting interface specific versions of confidence measures, to facilitate a comparison of confidence measures directly returned by the respective models. See Supplementary Figure S7 for more details. **(B)** Median ROC AUC of all analysed confidence measures.

In Figure 10A, we present the top-performing confidence measures for distinguishing experimentally enriched CD33-binding nanobodies from a background of candidate sequences derived from a camelid immunisation campaign against CD33, a target associated with acute myeloid leukaemia. The CD33 dataset differs fundamentally from the structure-derived benchmark: here, the negative sequences are realistic, experimentally observed immune repertoire sequences against the same antigen, rather than bait pairings from unrelated antigens. This makes the task harder and tests a more practically relevant discrimination.

In contrast to the structure-derived benchmark (Section 3.1), the overall ROC AUC values are substantially lower across all measures. In this analysis, the highest ROC AUC scores are achieved by AF3- and Chai-1-derived measures, whereas Boltz-2 and IntFold measures, which are strong for true-binder identification on the structure-derived benchmark, perform poorly. AF3 contributes the most high-scoring measures, while the single best measure is the pLDDT predicted by Chai-1, reaching a ROC AUC of 0.81. Further, we do not observe a clear bias towards globally or locally sensitive measures in the previous experiments. As the enriched binders form a small minority of the repertoire, we additionally report the PR-AUC (average precision). The ranking of confidence measures is largely preserved under PR-AUC (Spearman ρ=0.86 between the per-metric ROC AUC and PR-AUC) (Supplementary Figure S12; Supplementary Figure S13).

Overall, these results reinforce the conclusions from the previous sections: Finding a single confidence measure or model that performs well on structure-based discrimination tasks is not yet possible, and generalisation to new antigens and the complexity of real antibody distributions is limited.

## 4 Discussion

Performance is task-dependent: no single model or confidence measure dominates across both true binder identification and OOD detection, and we were not able to identify a clear mechanism explaining when or why a given model performs best. Our results show that Boltz-2 achieves the highest median performance for true binder identification for complexes from the RCSB Protein Database, although this advantage is not statistically significant given the limited number of antigens and the large inter-antigen variability. This shows that open-source options are a valid alternative to the AlphaFold model family. Importantly, the discriminative power of a model’s confidence measures and the structural accuracy of its predictions are distinct properties: Passaro et al. (2025) report that Boltz-2 lags behind AF3 on structure prediction in general, and specifically on antibody–antigen co-folding and generalisation to unseen antigens. Our finding that Boltz-2 confidence measures most consistently rank true nanobody binders highly should therefore not be read as Boltz-2 being categorically superior for nanobody modelling: the single best-ranked measures for this task are in fact IntFold’s Rosetta ΔG and pDockQ and AF3’s pDockQ2, so Boltz-2’s advantage lies in the consistency of its measures rather than in providing the top individual measure. However, across many antigens, all evaluated confidence measures struggle to rank the true binder above bait complexes, indicating that current structure prediction models do not reliably generalise to all nanobody–antigen pairs. This variability is not explained by simple factors such as antigen length or sequence similarity to known structures. We did, however, find that the structural confidence of the antigen-only prediction serves as a simple, largely model-agnostic indicator of how amenable an antigen is to structure-based screening, offering a practical, *a priori* estimate of an antigen’s targetability. Further physicochemical properties, such as epitope accessibility, glycosylation, antigen flexibility, and interface hydrophobicity, may additionally govern prediction difficulty and remain a promising avenue for future work.

Across Section 3.1, Section 3.2, and Section 3.3, a consistent pattern emerges: different tasks require different signals. Confidence measures capturing global and interface-level consistency, such as ipTM, perform better for true binder identification. This finding is consistent with prior findings by Teufel et al. (2023). On the other hand, local confidence measures and our novel measures specifically tailored to regions of interest, such as the CDR, are required to detect implausible or strongly perturbed sequences. This distinction is further supported by the alanine mutation analysis, where confidence measures sensitive to local perturbations or changes in the CDR track increasing mutation load, whereas many global confidence measures show lower correlation.

Rather than identifying a single best confidence measure, our results support candidate filtering by using multiple confidence measures in combination. Such approaches have also been explored by Vázquez Torres et al. (2025), who combine pAE, pLDDT, and Rosetta energy terms for candidate filtering. Our findings add nuance to this: the choice of confidence measures should depend on the candidate distribution, and in the case of nanobody sequences, special focus should be put on the CDRs. Furthermore, we found that confidence measure thresholds used in literature would discard up to 75% of real binding complexes, indicating a need for careful calibration.

The analysis of the CD33 dataset further highlights limitations in generalisation. In this realistic setting, where negatives are drawn from an experimentally observed immune repertoire rather than artificial bait pairings, overall performance drops substantially. Moreover, the clear distinction between globally and locally sensitive confidence measures observed in the structure-based benchmarks becomes less pronounced, indicating that real-world sequence distributions require both properties. These findings highlight a fundamental limitation: current confidence measures are primarily calibrated and benchmarked for structural plausibility rather than functional binding (Xu et al., 2025). As a result, they can rank geometrically consistent but non-binding complexes highly, while failing to distinguish subtle differences between realistic candidates.

Overall, the results suggest that future progress will require methods that go beyond structure confidence alone. While we already incorporate physics-based estimates of binding energetics, most prominently Rosetta ΔG , these are computed on the predicted structures rather than integrated into the structure prediction models themselves. Incorporating sequence-based signals or hybrid approaches combining structure prediction with learned scoring functions may further help to achieve robust performance across both synthetic benchmarks and realistic discovery datasets. Rather than the manual Pareto-front analysis used here, such signals could in principle be fused by a learned ensemble or by task-specific fine-tuning of a unified scoring function. We deliberately did not pursue this: with only 17 antigens and few confirmed binders per antigen, a learned combiner would be prone to overfitting and unlikely to generalise. We therefore leave learned ensembling and task-specific fine-tuning as future work, contingent on substantially larger ground-truth binding datasets.

### 4.1 Limitations and future work

Our analysis included statistical significance testing, yet only some pairwise comparisons reached significance and performance varied substantially across antigens. These observations are limited by the size of our benchmark: a substantially larger and more diverse test set might reveal clearer generalisation signal and more significant differences between measures, though we cannot establish this from the available data. The limited significance and the apparent difficulty in generalising across antigens may therefore partly reflect the modest benchmark size and high inter-antigen variability rather than a genuine absence of differences between measures.

The present study does not include experimental validation of predicted binding rankings; all conclusions are therefore limited to *in silico* performance. Relatedly, the non-binding labels in both the bait set and the CD33 negative set are assumptions: bait pairs are treated as non-binders because no binding is known, and unenriched CD33 repertoire sequences because they did not survive selection. Confirming the absence of weak or cross-reactive binding would require extensive additional wet-lab testing that was beyond the scope of this study, and undetected weak binders in the negative sets could affect the reported ROC AUC values. We further stress that the ROC AUC characterises how well a measure discriminates binders from non-binders independently of the baseline binding probability, but does not predict the absolute precision, and hence the experimental yield, attainable at a given prevalence; consistent with this, the complementary PR-AUC analysis shows low absolute precision on the rare binder class. This matches our prevalence-invariant screening question, whereas the expected yield in any concrete campaign will depend on the unknown deployment prevalence.

Our chosen dataset was carefully constructed to avoid training data leakage and to contain experimentally resolved nanobody–antigen structures. This small dataset allowed for a deeper analysis of multiple protein structure prediction models despite the high computing resources needed for such models and enabled analysis of structural effects under mutation. However, its small size reduces the statistical power of comparisons. Relaxing data constraints would allow the inclusion of larger datasets based on sequence-only binding information, representing a promising direction for future work. Likewise, datasets providing explicitly measured binding affinities, rather than only binary binding labels, would enable a finer-grained evaluation of confidence measures and are a worthwhile direction for future work.

pDockQ, and pDockQ2, and LIS were applied using the parameters reported in their original publications (Bryant et al., 2022; Zhu et al., 2023; Kim et al., 2024). Their performance may be improved by task-specific calibration, for example, by tuning on nanobody–antigen complexes deposited in the RCSB prior to 1 June 2023, for each structure prediction model.

Regarding sampling from protein structure prediction models, Abramson et al. (2024) report that prediction quality improves with the number of generated structures, whereas Bellinzona et al. (2024) find diminishing returns beyond a single replicate. Using five replicates per model and complex, as done in our analysis, represents a compromise between computational cost and robustness, but the optimal number of replicates likely depends on the specific application.

## Data Availability

In this study, publicly available data for the RCSB Protein Data Bank (Burley et al., 2021) were used for the analysis.

## References

[B1] Abanades B. Georges G. Bujotzek A. Deane C. M. (2022a). ABlooper: fast accurate antibody CDR loop structure prediction with accuracy estimation. Bioinformatics 38, 1877–1880. 10.1093/bioinformatics/btac016 PMC8963302 35099535

[B2] Abanades B. Wong W. K. Boyles F. Georges G. Bujotzek A. Deane C. M. (2022b). Immunebuilder: Deep-Learning Models for Predicting the Structures of Immune Proteins. 10.1101/2022.11.04.514231 PMC10227038 37248282

[B3] Abbasi W. A. Yaseen A. Hassan F. U. Andleeb S. Minhas F. U. A. A. (2020). ISLAND: *in-silico* proteins binding affinity prediction using sequence information. BioData Min. 13, 20. 10.1186/s13040-020-00231-w 33292419 PMC7688004

[B4] Abramson J. Adler J. Dunger J. Evans R. Green T. Pritzel A. (2024). Accurate structure prediction of biomolecular interactions with AlphaFold 3. Nature 630, 493–500. 10.1038/s41586-024-07487-w PMC11168924 38718835

[B5] Alford R. F. Leaver-Fay A. Jeliazkov J. R. O’Meara M. J. DiMaio F. P. Park H. (2017). The rosetta all-atom energy function for macromolecular modeling and design. J. Chem. Theory Comput. 13, 3031–3048. 10.1021/acs.jctc.7b00125 28430426 PMC5717763

[B6] Baek M. DiMaio F. Anishchenko I. Dauparas J. Ovchinnikov S. Lee G. R. (2021). Accurate prediction of protein structures and interactions using a three-track neural network. Science 373, 871–876. 10.1126/science.abj8754 PMC7612213 34282049

[B7] Baek M. McHugh R. Anishchenko I. Jiang H. Baker D. DiMaio F. (2024). Accurate prediction of protein–nucleic acid complexes using RoseTTAFoldNA. Nat. Methods 21, 117–121. 10.1038/s41592-023-02086-5 37996753 PMC10776382

[B8] Basu S. Wallner B. (2016). DockQ: a quality measure for protein-protein docking models. PLOS ONE 11, e0161879. 10.1371/journal.pone.0161879 27560519 PMC4999177

[B9] Bellinzona G. Sassera D. Bonvin A. M. J. J. (2024). Accelerating protein–protein interaction screens with reduced AlphaFold-Multimer sampling. Bioinforma. Adv. 4, vbae153. 10.1093/bioadv/vbae153 39464748 PMC11513016

[B10] Bennett N. R. Coventry B. Goreshnik I. Huang B. Allen A. Vafeados D. (2023). Improving *de novo* protein binder design with deep learning. Nat. Commun. 14, 2625. 10.1038/s41467-023-38328-5 PMC10163288 37149653

[B11] Bernacki J. P. Murphy R. M. (2011). Length-dependent aggregation of uninterrupted polyalanine peptides. Biochemistry 50, 9200–9211. 10.1021/bi201155g 21932820

[B12] Bryant P. Pozzati G. Elofsson A. (2022). Improved prediction of protein-protein interactions using AlphaFold2. Nat. Commun. 13, 1265. 10.1038/s41467-022-28865-w 35273146 PMC8913741

[B13] Buel G. R. Walters K. J. (2022). Can AlphaFold2 predict the impact of missense mutations on structure? Nat. Struct. and Mol. Biol. 29, 1–2. 10.1038/s41594-021-00714-2 PMC11218004 35046575

[B14] Burley S. K. Bhikadiya C. Bi C. Bittrich S. Chen L. Crichlow G. V. (2021). RCSB protein data bank: powerful new tools for exploring 3D structures of biological macromolecules for basic and applied research and education in fundamental biology, biomedicine, biotechnology, bioengineering and energy sciences. Nucleic Acids Res. 49, D437–D451. 10.1093/nar/gkaa1038 33211854 PMC7779003

[B15] Chai Discovery Boitreaud J. Dent J. McPartlon M. Meier J. Reis V. (2024). Chai-1: Decoding the molecular interactions of life. 10.1101/2024.10.10.615955

[B16] Chai Discovery Boitreaud J. Chen R. Dent J. Fairweather L. Geisz D. (2025). Drug-like antibody design against challenging targets with atomic precision. 10.1101/2025.11.29.691346

[B17] Chakraborty S. Venkatramani R. Rao B. J. Asgeirsson B. Dandekar A. M. (2013). Protein structure quality assessment based on the distance profiles of consecutive backbone C*α* atoms. F1000Research 2, 211. 10.12688/f1000research.2-211.v3 24555103 PMC3892923

[B18] Cowen-Rivers A. I. Gorinski P. J. Sootla A. Khan A. Furui L. Wang J. (2022). Structured q-learning for antibody design. arXiv Preprint arXiv:2209.04698. 10.48550/ARXIV.2209.04698

[B19] Darling A. L. Uversky V. N. (2017). Intrinsic disorder in proteins with pathogenic repeat expansions. Mol. A J. Synthetic Chem. Nat. Prod. Chem. 22, 2027. 10.3390/molecules22122027 29186753 PMC6149999

[B20] Dauparas J. Anishchenko I. Bennett N. Bai H. Ragotte R. J. Milles L. F. (2022). Robust deep learning–based protein sequence design using ProteinMPNN. Science 378, 49–56. 10.1126/science.add2187 PMC9997061 36108050

[B21] Dunbar J. Deane C. M. (2016). ANARCI: antigen receptor numbering and receptor classification. Bioinformatics 32, 298–300. 10.1093/bioinformatics/btv552 26424857 PMC4708101

[B22] Dunbrack R. L. (2025). RēS Ipsae Loquunt: What’s Wrong with Alphafold’s Iptm Score and How to Fix It. 10.1101/2025.02.10.637595

[B23] Evans R. O’Neill M. Pritzel A. Antropova N. Senior A. Green T. (2022). Protein Complex Prediction with AlphaFold-Multimer. 10.1101/2021.10.04.463034

[B24] Hernandez R. Facelli J. C. (2022). Structure analysis of the proteins associated with PolyA repeat expansion disorders. J. Biomolecular Structure and Dynamics 40, 5556–5565. 10.1080/07391102.2021.1871957 33459170 PMC8286276

[B25] Hitawala F. N. Gray J. J. (2025). What does AlphaFold3 learn about antibody and nanobody docking, and what remains unsolved?. mAbs. 17 (1). 10.1080/19420862.2025.2545601 40814020 PMC12360200

[B26] Høie M. H. Gade F. S. Johansen J. M. Würtzen C. Winther O. Nielsen M. (2024). DiscoTope-3.0: improved B-cell epitope prediction using inverse folding latent representations. Front. Immunol. 15, 1322712. 10.3389/fimmu.2024.1322712 PMC10882062 38390326

[B27] Hýsková A. Maršálková E. Šimeček P. (2026). Balancing speed and precision in protein folding: a comparison of AlphaFold2, ESMFold, and OmegaFold. Front. Genet. 16, 1715037. 10.3389/fgene.2025.1715037 PMC12844563 41608648

[B28] Jendrusch M. A. Yang A. L. J. Cacace E. Bobonis J. Voogdt C. G. P. Kaspar S. (2025). AlphaDesign: a *de novo* protein design framework based on AlphaFold. Mol. Syst. Biol. 21, 1166–1189. 10.1038/s44320-025-00119-z PMC12405559 40527958

[B29] Jumper J. Evans R. Pritzel A. Green T. Figurnov M. Ronneberger O. (2021). Highly accurate protein structure prediction with AlphaFold. Nature 596, 583–589. 10.1038/s41586-021-03819-2 34265844 PMC8371605

[B30] Khan M. A. Cowen-Rivers A. I. Deik D. Grosnit A. Dreczkowski K. Robert P. A. (2023). Towards real-world automated antibody design with combinatorial bayesian optimisation. Cell Rep. Method, 3 (1), 100374. 10.1016/j.crmeth.2022.100374 36814835 PMC9939385

[B31] Kim A.-R. Hu Y. Comjean A. Rodiger J. Mohr S. E. Perrimon N. (2024). Enhanced Protein-Protein Interaction Discovery via AlphaFold-Multimer. 10.1101/2024.02.19.580970

[B32] Kosugi T. Ohue M. (2022). Solubility-aware protein binding peptide design using AlphaFold. Biomedicines 10, 1626. 10.3390/biomedicines10071626 35884931 PMC9312799

[B33] Lefranc M.-P. Pommié C. Ruiz M. Giudicelli V. Foulquier E. Truong L. (2003). IMGT unique numbering for immunoglobulin and T cell receptor variable domains and Ig superfamily v-like domains. Dev. Comp. Immunol. 27, 55–77. 10.1016/s0145-305x(02)00039-3 12477501

[B34] Lin Z. Akin H. Rao R. Hie B. Zhu Z. Lu W. (2023). Evolutionary-scale prediction of atomic-level protein structure with a language model. Science 379, 1123–1130. 10.1126/science.ade2574 36927031

[B35] Liu G. Zeng H. Mueller J. Carter B. Wang Z. Schilz J. (2020). Antibody complementarity determining region design using high-capacity machine learning. Bioinformatics 36, 2126–2133. 10.1093/bioinformatics/btz895 31778140 PMC7141872

[B36] Liu L. Zhang S. Xue Y. Ye X. Zhu K. Li Y. (2024). Technical Report of HelixFold3 for Biomolecular Structure Prediction. 10.48550/arXiv.2408.16975

[B37] Luo S. Su Y. Peng X. Wang S. Peng J. Ma J. (2022). Antigen-specific antibody design and optimization with diffusion-based generative models for protein structures. Adv. Neural Inf. Process. Syst. 9754–9767. Available online at: https://openreview.net/forum?id=jSorGn2Tjg.

[B38] Melnyk I. Lozano A. Das P. Chenthamarakshan V. (2025). AlphaFold distillation for inverse protein design. Sci. Rep. 15, 21743. 10.1038/s41598-025-00436-1 40595650 PMC12216305

[B39] Morrison K. L. Weiss G. A. (2001). Combinatorial alanine-scanning. Curr. Opin. Chem. Biol. 5, 302–307. 10.1016/S1367-5931(00)00206-4 11479122

[B40] Norman R. A. Ambrosetti F. Bonvin A. M. J. J. Colwell L. J. Kelm S. Kumar S. (2020). Computational approaches to therapeutic antibody design: established methods and emerging trends. Briefings Bioinforma. 21, 1549–1567. 10.1093/bib/bbz095 31626279 PMC7947987

[B41] Pak M. A. Markhieva K. A. Novikova M. S. Petrov D. S. Vorobyev I. S. Maksimova E. S. (2021). Using Alphafold to Predict the Impact of Single Mutations on Protein Stability and Function. 10.1101/2021.09.19.460937 PMC10019719 36928239

[B42] Passaro S. Corso G. Wohlwend J. Reveiz M. Thaler S. Somnath V. R. (2025). Boltz-2: Towards Accurate and Efficient Binding Affinity Prediction. 10.1101/2025.06.14.659707

[B43] Protenix Team Zhang Y. Gong C. Zhang H. Ma W. Liu Z. (2026). Protenix-v1: Toward High-Accuracy Open-Source Biomolecular Structure Prediction. 10.64898/2026.02.05.703733

[B44] Romero-Molina S. Ruiz-Blanco Y. B. Mieres-Perez J. Harms M. Münch J. Ehrmann M. (2022). PPI-Affinity: a web tool for the prediction and optimization of protein–peptide and protein–protein binding affinity. J. Proteome Res. 21, 1829–1841. 10.1021/acs.jproteome.2c00020 PMC9361347 35654412

[B45] Ruffolo J. A. Chu L.-S. Mahajan S. P. Gray J. J. (2022). Fast, Accurate Antibody Structure Prediction from Deep Learning on Massive Set of Natural Antibodies. 10.1101/2022.04.20.488972 PMC10129313 37185622

[B46] Teufel F. Refsgaard J. C. Kasimova M. A. Deibler K. Madsen C. T. Stahlhut C. (2023). Deorphanizing peptides using structure prediction. J. Chem. Inf. Model. 63, 2651–2655. 10.1021/acs.jcim.3c00378 37092865

[B47] The IntFold Team Qiao L. Bai W. Yan H. Liu G. Xi N. (2025). Intfold: A Controllable Foundation Model for General and Specialized Biomolecular Structure Prediction. 10.48550/arXiv.2507.02025

[B48] Vangone A. Bonvin A. M. J. J. (2017). PRODIGY: a contact-based predictor of binding affinity in protein-protein complexes. Bio-protocol 7, e2124. 10.21769/BioProtoc.2124 34458447 PMC8376549

[B49] Vázquez Torres S. Benard Valle M. Mackessy S. P. Menzies S. K. Casewell N. R. Ahmadi S. (2025). *De novo* designed proteins neutralize lethal snake venom toxins. Nature 639, 225–231. 10.1038/s41586-024-08393-x 39814879 PMC11882462

[B50] Wang J. Lisanza S. Juergens D. Tischer D. Watson J. L. Castro K. M. (2022). Scaffolding protein functional sites using deep learning. Science 377, 387–394. 10.1126/science.abn2100 PMC9621694 35862514

[B51] Wang Y. Lu J. Jaitly N. Susskind J. Bautista M. A. (2025). Simplefold: Folding Proteins Is Simpler than You Think. 10.48550/arXiv.2509.18480

[B52] Watson J. L. Juergens D. Bennett N. R. Trippe B. L. Yim J. Eisenach H. E. (2023). *De novo* design of protein structure and function with RFdiffusion. Nature 620, 1089–1100. 10.1038/s41586-023-06415-8 37433327 PMC10468394

[B53] Wohlwend J. Corso G. Passaro S. Reveiz M. Leidal K. Swiderski W. (2024). Boltz-1 Democratizing Biomolecular Interaction Modeling. 10.1101/2024.11.19.624167

[B54] Wohlwend J. Reveiz M. McPartlon M. Feldmann A. Jin W. Barzilay R. (2025). MiniFold: simple, fast, and accurate protein structure prediction. Trans. Mach. Learn. Res.

[B55] Wu R. Ding F. Wang R. Shen R. Zhang X. Luo S. (2022). High-resolution De Novo Structure Prediction from Primary Sequence. 10.1101/2022.07.21.500999

[B56] Xu J. L. Davis M. M. (2000). Diversity in the CDR3 region of VH is sufficient for Most antibody specificities. Immunity 13, 37–45. 10.1016/S1074-7613(00)00006-6 10933393

[B57] Xu S. Feng Q. Qiao L. Wu H. Shen T. Cheng Y. (2025). Benchmarking all-atom biomolecular structure prediction with FoldBench. Nat. Commun. 17, 442. 10.1038/s41467-025-67127-3 41345395 PMC12800276

[B58] Yang Y. X. Huang J. Y. Wang P. Zhu B. T. (2023). AREA-AFFINITY: a web server for machine learning-based prediction of protein–protein and antibody–protein antigen binding affinities. J. Chem. Inf. Model. 63, 3230–3237. 10.1021/acs.jcim.2c01499 37235532 PMC10268951

[B59] Yin R. Pierce B. G. (2024). Evaluation of AlphaFold antibody-antigen modeling with implications for improving predictive accuracy. Protein Sci. A Publ. Protein Soc. 33, e4865. 10.1002/pro.4865 38073135 PMC10751731

[B60] Zhou X. Xue D. Chen R. Zheng Z. Wang L. Gu Q. (2024). “Antigen-specific antibody design *via* direct energy-based preference optimization,” in The thirty-eighth Annual Conference on Neural Information Processing Systems.

[B61] Zhou Y. Lu C. Ma Y. Qu W. Ye F. Zhang K. (2025). Seedfold: Scaling Biomolecular Structure Prediction. 10.48550/arXiv.2512.24354

[B62] Zhu W. Shenoy A. Kundrotas P. Elofsson A. (2023). Evaluation of AlphaFold-Multimer prediction on multi-chain protein complexes. Bioinformatics 39, btad424. 10.1093/bioinformatics/btad424 PMC10348836 37405868

