# Correction: Analysing open-source protein folding models for nanobody binding prediction

**DOI:** 10.3389/fbinf.2026.1988897

**Published:** 2026-09-25

**Authors:** Yannick Vogt, Rebekka Roßberg, Jan Habermann, Pia Kluge, Wendan Xu, Mehdi Naouar, Stefan Hirschberg, Cornelius Miething, Joschka Bödecker, Maria Kalweit, Evelyn Ullrich, Gabriel Kalweit

**Affiliations:** 1 Department of Computer Science, University of Freiburg, Freiburg, Germany; 2 Collaborative Research Institute Intelligent Oncology (CRIION), Freiburg, Germany; 3 Experimental Immunology and Cell Therapy, Department of Pediatrics, Goethe University, Frankfurt am Main, Germany; 4 Department of Medicine I, University Medical Center Freiburg, University of Freiburg, Freiburg, Germany; 5 German Cancer Consortium (DKTK), Partner Site Freiburg, Freiburg, Germany; 6 preclinics certified products GmbH, Potsdam, Germany; 7 preclinics Gesellschaft für präklinische Forschung mbH, Potsdam, Germany; 8 Intelligent Machine-Brain Interfacing Technology (IMBIT), BrainLinks-BrainTools, University of Freiburg, Freiburg, Germany; 9 German Cancer Consortium (DKTK), Partner Site Frankfurt/Mainz, Frankfurt am Main, Germany

**Keywords:** AlphaFold3, binding prediction, Boltz-2, Chai-1, confidence measure, *In silico* screening, nanobody, IntFold

The author contributions statement was erroneously given as:

YV: Data curation, Formal Analysis, Investigation, Methodology, Software, Validation, Visualization, Writing – original draft, Writing – review and editing. RR: Data curation, Formal Analysis, Investigation, Methodology, Software, Validation, Visualization, Writing – original draft, Writing – review and editing. JH: Data curation, Investigation, Validation, Writing – original draft, Writing – review and editing. PK: Data curation, Investigation, Validation, Writing – original draft, Writing – review and editing. WX: Validation, Writing – original draft, Writing – review and editing. MN: Validation, Visualization, Writing – original draft, Writing – review and editing. SH: Data curation, Investigation, Resources, Writing – original draft, Writing – review and editing. CM: Conceptualization, Funding acquisition, Writing – original draft, Writing – review and editing. JB: Conceptualization, Funding acquisition, Writing – original draft, Writing – review and editing. MK: Conceptualization, Funding acquisition, Project administration, Resources, Supervision, Validation, Writing – original draft, Writing – review and editing. EU: Conceptualization, Funding acquisition, Investigation, Resources, Validation, Writing – original draft, Writing – review and editing. GK: Conceptualization, Funding acquisition, Supervision, Validation, Writing – original draft, Writing – review and editing.

The correct **author contributions** statement is:

YV: Data curation, Formal Analysis, Investigation, Methodology, Software, Validation, Visualization, Writing – original draft, Writing – review and editing. RR: Data curation, Formal Analysis, Investigation, Methodology, Software, Validation, Visualization, Writing – original draft, Writing – review and editing. JH: Data curation, Investigation, Validation, Writing – original draft, Writing – review and editing. PK: Data curation, Investigation, Validation, Writing – original draft, Writing – review and editing. WX: Validation, Writing – original draft, Writing – review and editing. MN: Validation, Visualization, Writing – original draft, Writing – review and editing. SH: Data curation, Investigation, Resources, Validation, Writing – original draft, Writing – review and editing. CM: Conceptualization, Funding acquisition, Writing – original draft, Writing – review and editing. JB: Conceptualization, Funding acquisition, Resources, Writing – original draft, Writing – review and editing. MK: Conceptualization, Funding acquisition, Project administration, Resources, Supervision, Validation, Writing – original draft, Writing – review and editing. EU: Conceptualization, Funding acquisition, Investigation, Resources, Validation, Writing – original draft, Writing – review and editing. GK: Conceptualization, Funding acquisition, Supervision, Validation, Writing – original draft, Writing – review and editing.

The conflict of interest statement was erroneously given as

Author SH was employed by Preclinics certified products GmbH.

The remaining author(s) declared that this work was conducted in the absence of any commercial or financial relationships that could be construed as a potential conflict of interest. The author EU declared that they were an editorial board member of Frontiers at the time of submission. This had no impact on the peer review process and the final decision.

The correct **conflict of interest** statement is

Author SH was employed by preclinics certified products GmbH.

EU has a sponsored research project with Gilead and BMS and acts as medical advisor of Phialogics and CRIION.

The remaining author(s) declared that this work was conducted in the absence of any commercial or financial relationships that could be construed as a potential conflict of interest. The author EU declared that they were an editorial board member of Frontiers at the time of submission. This had no impact on the peer review process and the final decision.

The references for Chai et al., 2024 and Chai et al., 2025 were erroneously written as Chai, D., Boitreaud, J., Dent, J., McPartlon, M., Meier, J., Reis, V., et al. (2024). Chai-1: Decoding the Molecular Interactions of Life. doi: 10.1101/2024.10.10.615955 and Chai, D., Boitreaud, J., Chen, R., Dent, J., Fairweather, L., Geisz, D., et al. (2025). Drug-Like Antibody Design Against Challenging Targets with Atomic Precision. doi: 10.1101/2025.11.29.691346.

It should be Chai Discovery, Boitreaud, J., Dent, J., McPartlon, M., Meier, J., Reis, V., et al. (2024). Chai-1: Decoding the Molecular Interactions of Life. doi: 10.1101/2024.10.10.615955 and Chai Discovery, Boitreaud, J., Chen, R., Dent, J., Fairweather, L., Geisz, D., et al. (2025). Drug-Like Antibody Design Against Challenging Targets with Atomic Precision. doi: 10.1101/2025.11.29.691346.

The reference (Hitawala and Gray, 2024) was erroneously cited by its preprint titled “What has AlphaFold3 learned about antibody and nanobody docking, and what remains unsolved?” published in bioRxiv in 2024. It should be (Hitawala and Gray, 2025) titled “What does AlphaFold3 learn about antibody and nanobody docking, and what remains unsolved?” published in mAbs in 2025.

The original version of this article has been updated.

