# Supplementary material for: Analysing open-source protein folding models for nanobody binding prediction
Source: Front Bioinform. 2026 Jul 22;6:1858891. doi: 10.3389/fbinf.2026.1858891 (PMC13437531; doi:10.3389/fbinf.2026.1858891)
Supplement: Supplementary file 1 [file DataSheet1.pdf]

Supplementary Material

0.1 Implementation details

The interface predicted TM Score based on Aligned Errors (ipSAE) measure only considers residue-pairs with a predicted Alignment Error (pAE) below a given threshold. For our analysis, we tested pAE thresholds of 10, 20, and 100. We found 20 to perform best and report on this version in our results.

For the alanine scanning analysis, a total of 4 complexes had to be excluded as several intermediate mutation variants were not recognised as valid nanobodies by ANARCI (Dunbar and Deane, 2016), preventing computation of all CDR-specific measures, which led to an exclusion from the alanine scanning experiments.

For IntFold, we use predicted Local Distance Difference Test (pLDDT) computed from Cα atoms only, for consistency with the other models. The IntFold Team et al. (2025) state in Section 4.2 that the jackhmmer software (as in AlphaFold3 (AF3)) was used to generate Multiple Sequence Alignment (MSA)s for paired sequences and ColabFold (Mirdita et al., 2022) otherwise. However, the public repository uses only ColabFold, as in Boltz-2, so we used ColabFold for our experiments.

For all evaluated models (AF3, Boltz-2, Chai-1, and IntFold), we used the prediction hyperparameters exactly as specified by each model's default configuration, with no task-specific adaptation; this includes the number of recycles, the diffusion and sampling settings, and the MSA generation, for which each model's own default pipeline was used. The only settings we fixed consistently across models were a single global random seed and the number of generated structures, namely five samples per nanobody–antigen complex and model. For each complex, we report the confidence measures of the single sample ranked highest by that model's own aggregate ranking score (the *aggr. score*).

0.2 Confidence Measures and Details for Predicting True Binders

In Figure S1 we present the Receiver Operating Characteristic Area Under the Curve (ROC AUC) for all 60 analysed confidence measures when identifying true binder complexes. See Figure S2 for a pairwise significance analysis of all metrics. Here, a p-value below 0.05 indicates that a confidence measure had a significantly higher ROC AUC across all 17 analysed antigens.

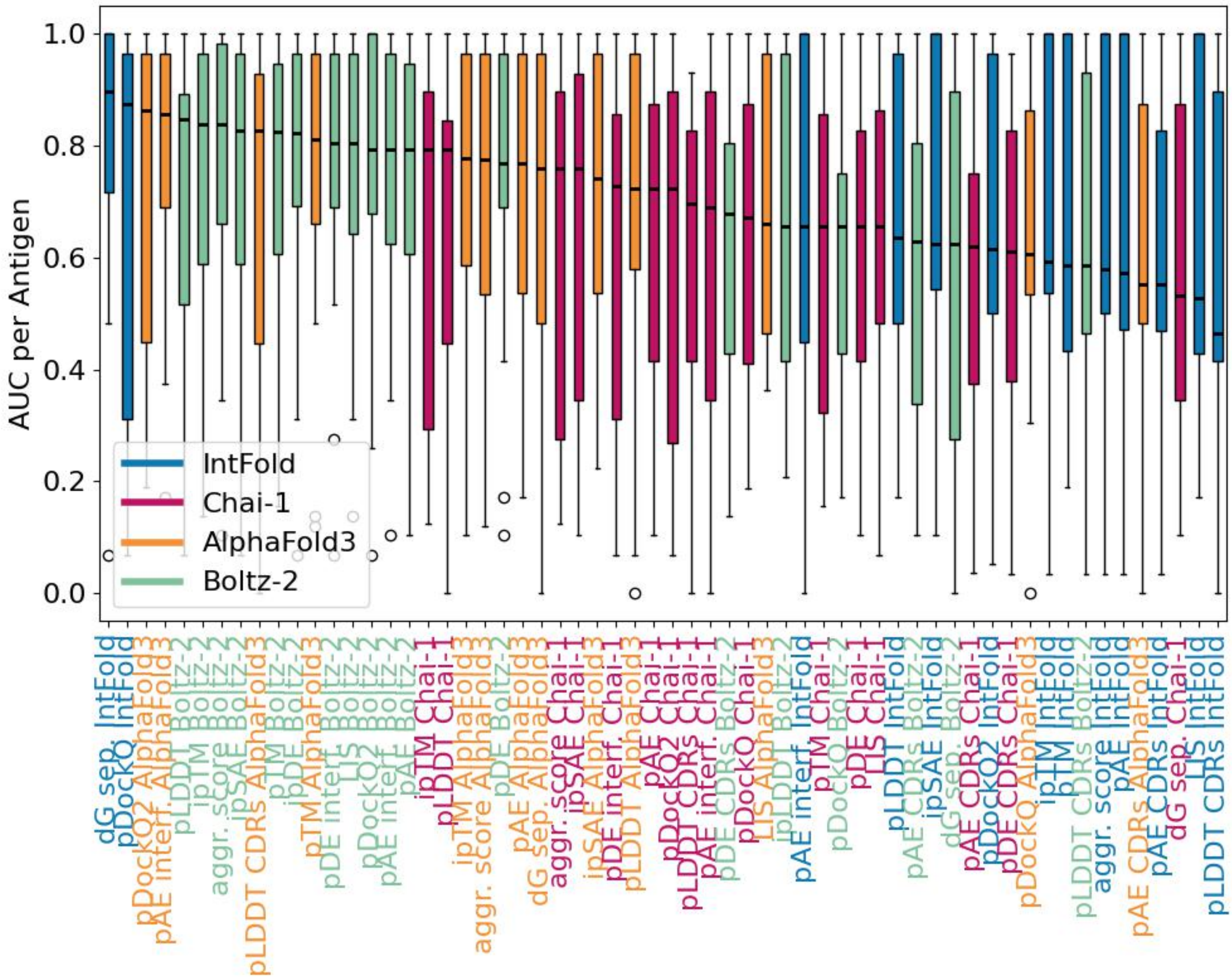

Figure S1. True Binder Detection: Boxplot of ROC AUC for all analysed confidence measures when identifying true binder complexes.

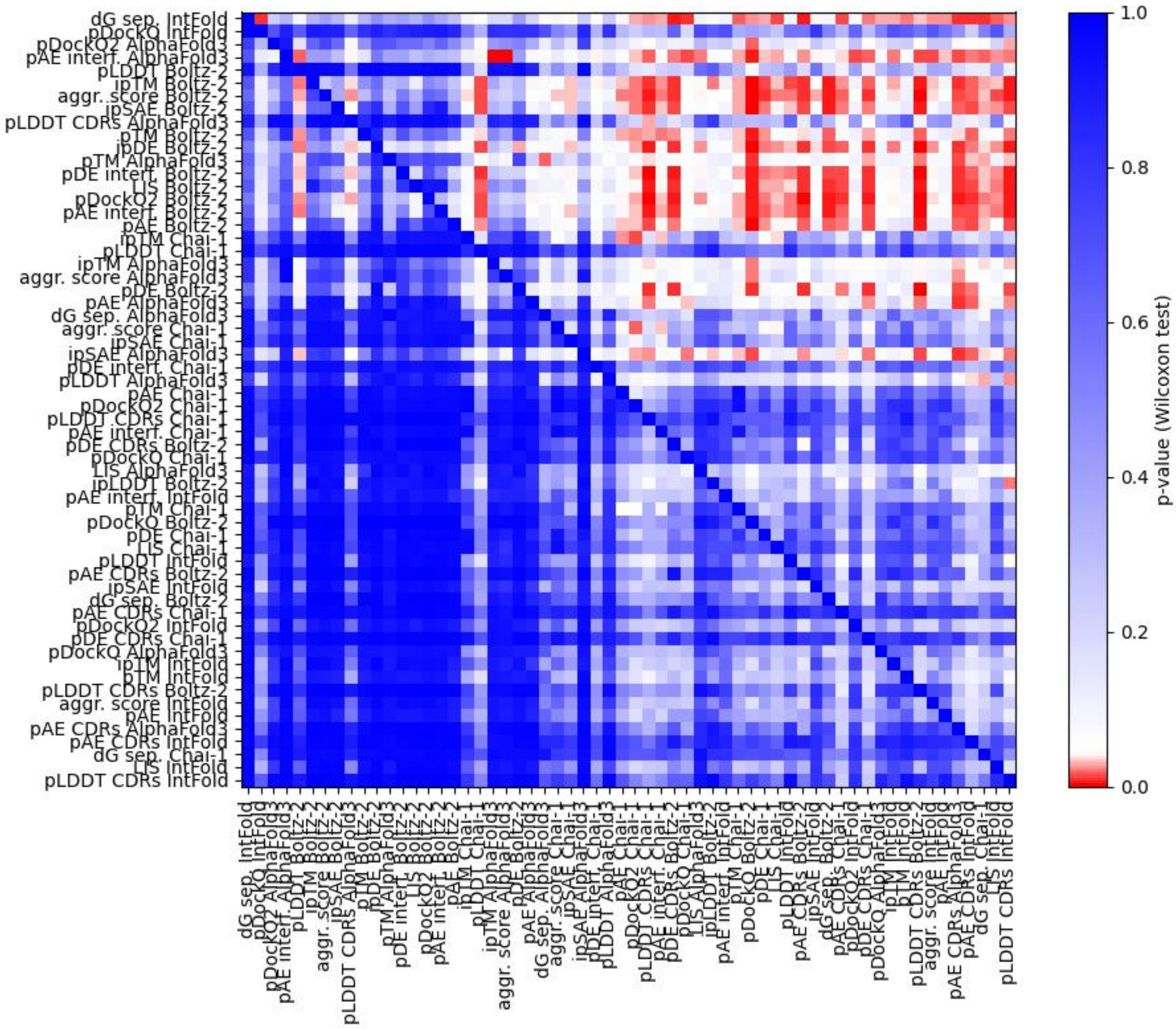

Figure S2. True Binder Detection: Heatmap of p-values for significant analysis between all 60 predicted confidence measures. Red colouring indicates when a measure on the y-axis achieves a significantly higher ROC AUC than a measure it was compared to on the y-axis.

0.3 Confidence Measures and Details for Identifying Out-Of-Distribution (OOD) Sequences

In Figure S3 we present the ROC AUC for all 60 analysed confidence measures when identifying OOD sequences. See Figure S4 for a pairwise significance analysis of all metrics. Here, a p-value below 0.05 indicates that a confidence measure had a significantly higher ROC AUC across all 17 analysed antigens.

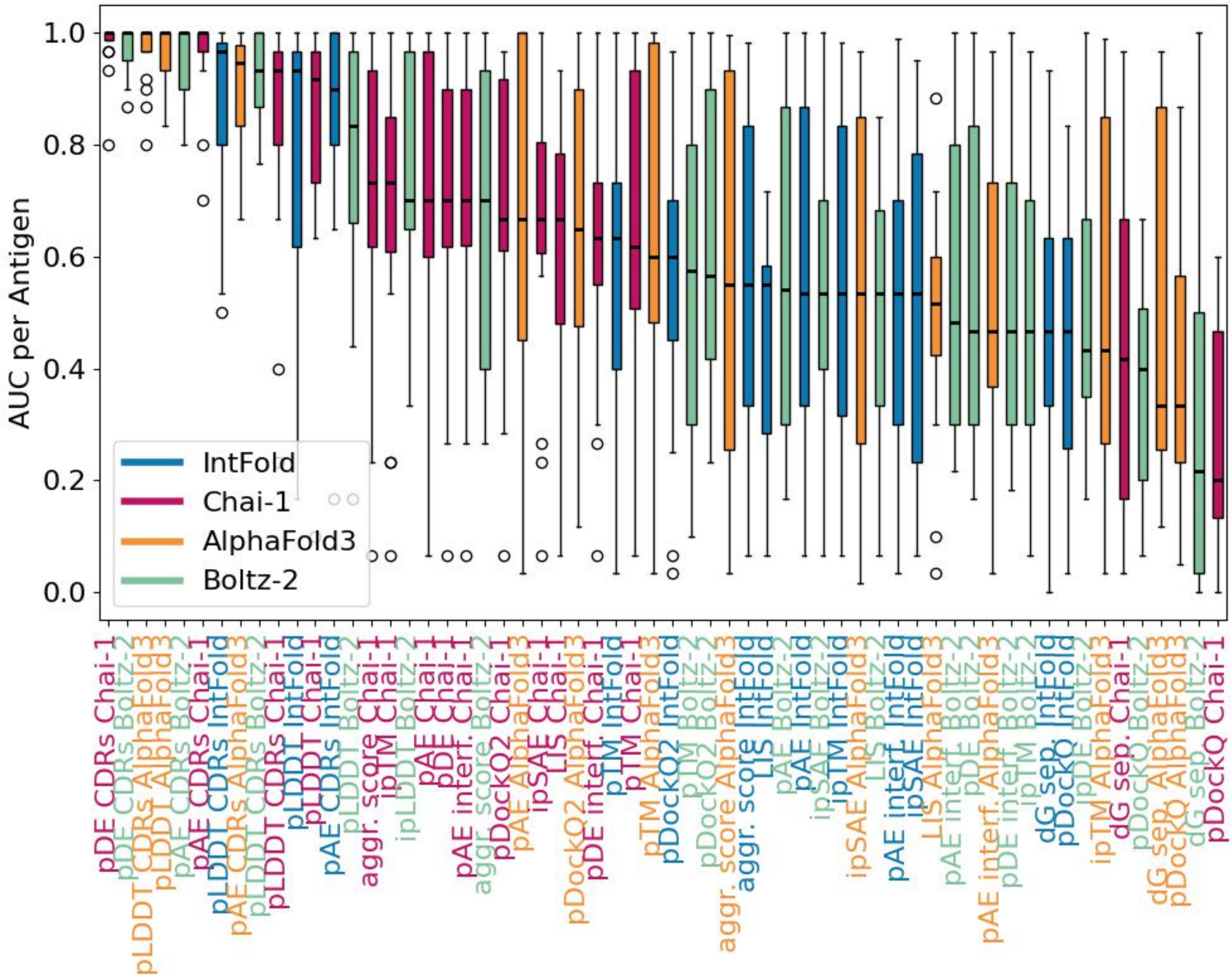

Figure S3. OOD Sequence Detection: Boxplot of ROC AUC for all analysed confidence measures when identifying OOD sequences.

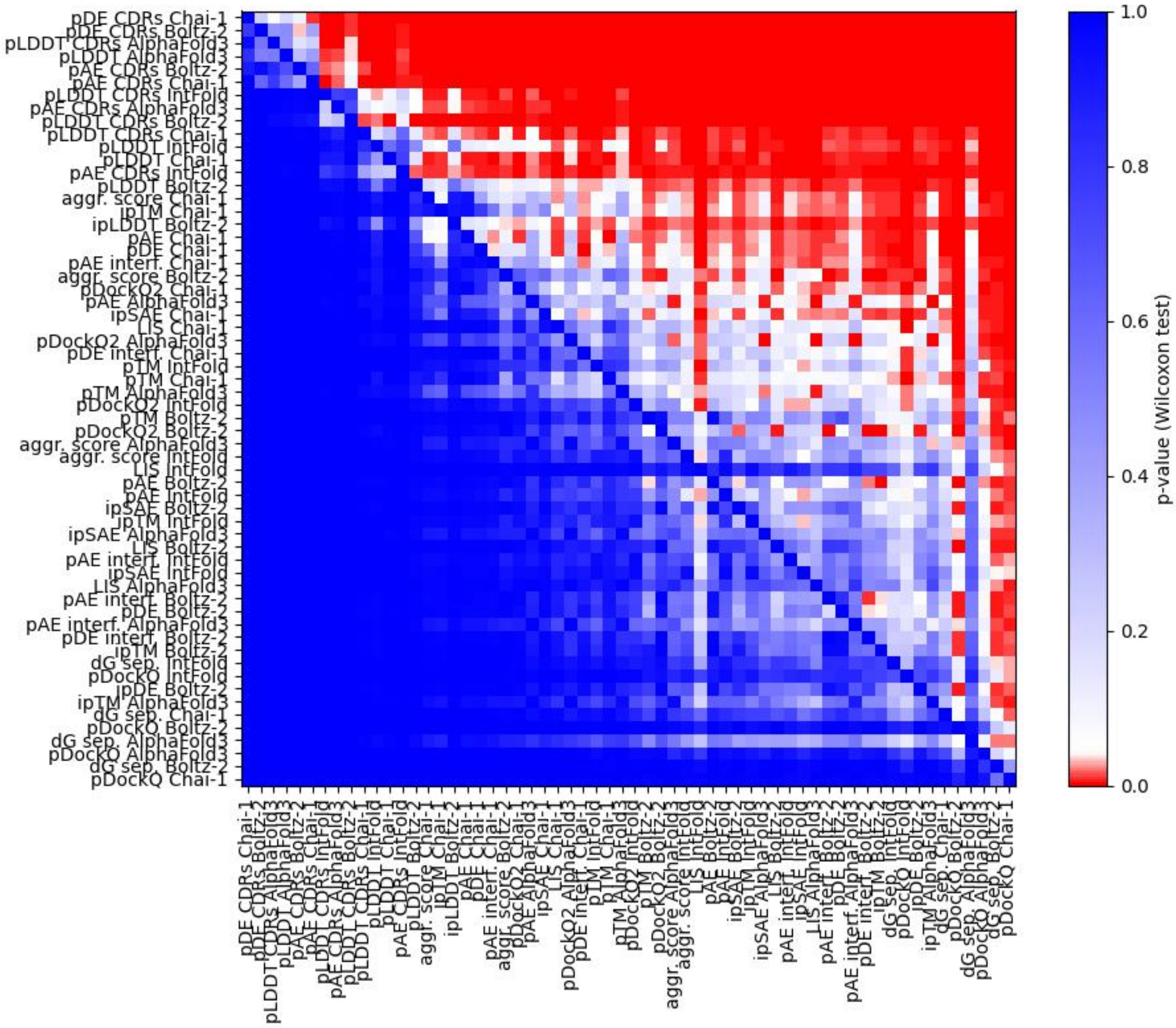

Figure S4. OOD Sequence Detection: Heatmap of p-values for significant analysis between all 60 predicted confidence measures. Red colouring indicates when a measure on the y-axis achieves a significantly higher ROC AUC than a measure it was compared to on the y-axis.

0.4 Significance of Correlation in Alanine Scanning

In the results, we presented the correlation between confidence measure change and the amount of mutations introduced in the Complementarity Determining Region (CDR)3. However, not all these correlations were statistically significant. In Figure S5, we present the fraction of statistically significant correlations, over 13 antigens, per confidence measure.

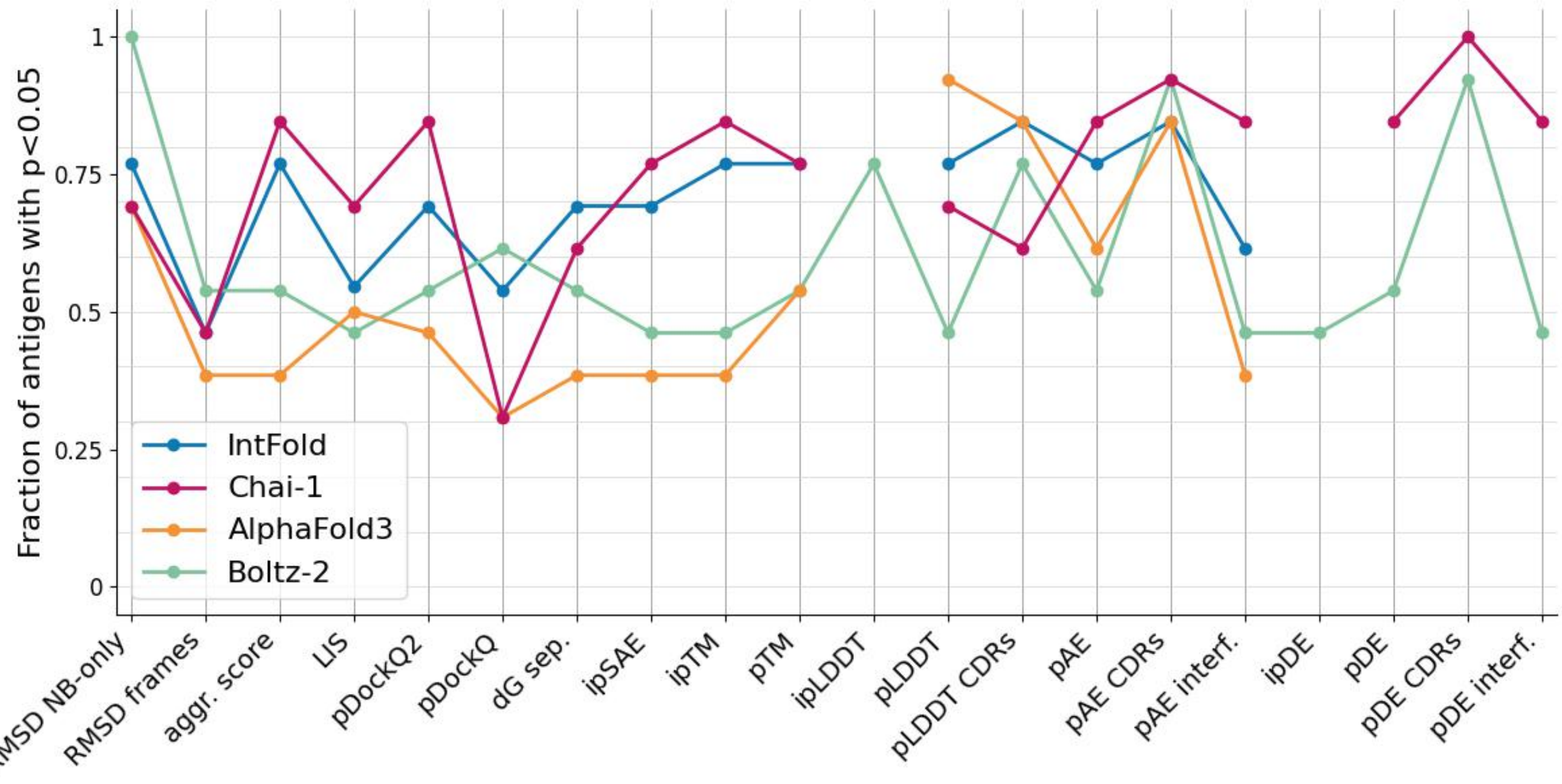

Figure S5. Fraction of antigens with a statistically significant correlation between the number of alanine mutations and measurement change.

0.5 Antigen Structure Stability under CDR3 Alanine Scanning

In the main text we analyse how the predicted *nanobody* structure responds to progressive CDR3 alanine mutation (Figure 6). Here we instead ask whether the predicted *antigen* changes. For every increasing-alanine prediction we reduce the complex to the antigen chain, superimpose it onto the *experimentally determined* antigen of the same complex (antigen-on-antigen, without aligning on the nanobody), and report the resulting Cα-Root Mean Square Distance (RMSD), shown in Figure S6(A). As the antigen sequence is identical across all mutation levels, a curve that grows with the number of mutations indicates that the predictor repositions or distorts the antigen, away from its real conformation, as the binder is mutated. The value at zero mutations is the antigen prediction error of the unmutated complex.

Interestingly, one antigen, 9GIX, behaves markedly differently from the rest: its predicted antigen drifts strongly from the experimental structure as alanine mutations accumulate in the corresponding nanobody (Cα-RMSD rising from about 3.7Å to about 11Å), whereas for all other antigens the antigen prediction remains essentially stable. Notably, 9GIX is at the same time one of the antigens whose *nanobody* structure changes the least under the same mutations (Figure 6, nanobody Cα-RMSD staying around 2.4Å). The model thus appears to absorb the disruptive sequence change predominantly in the antigen rather than in the mutated nanobody for this complex, as illustrated in Figure S6(B–D).

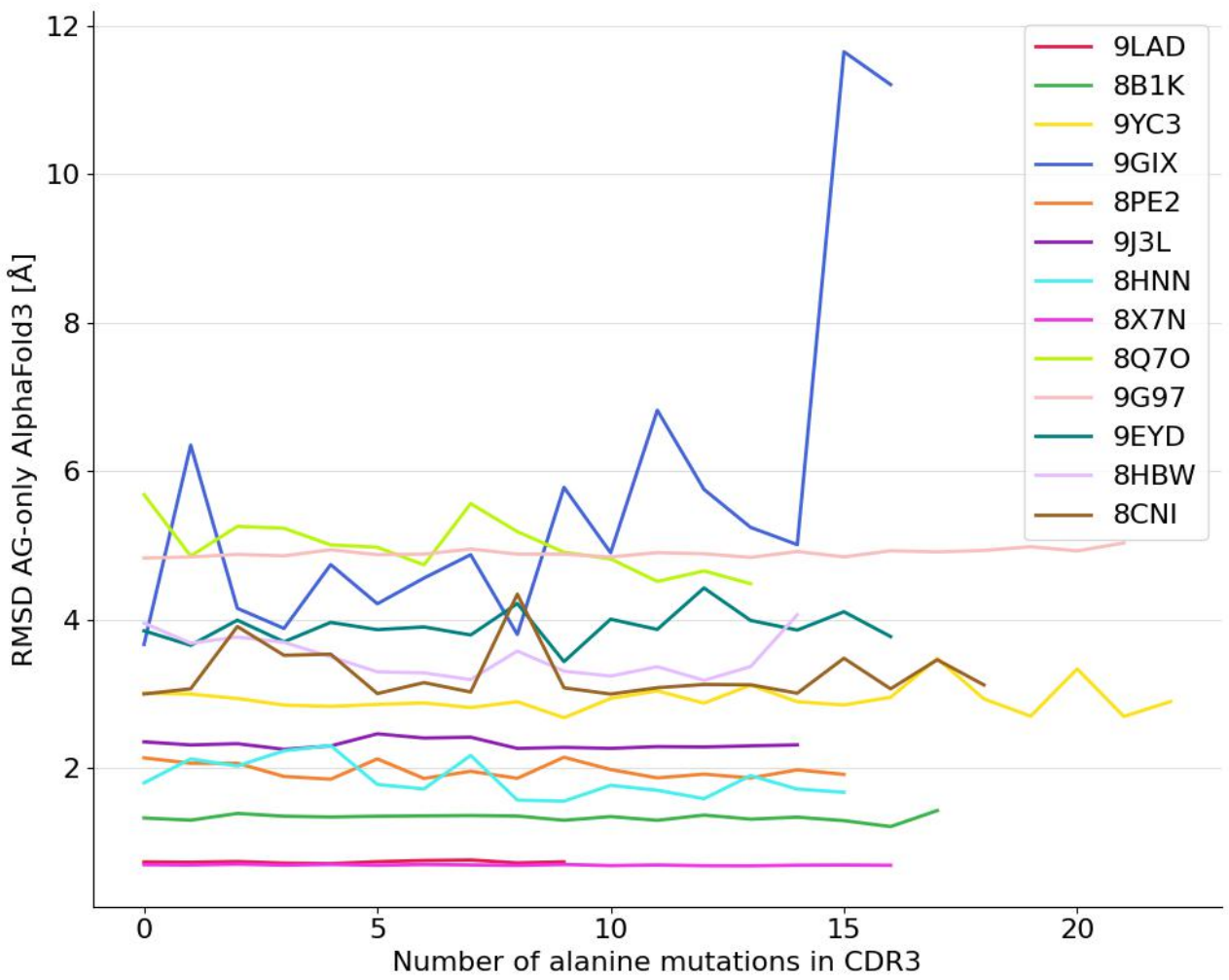

(A) Antigen Cα-RMSD vs. experiment.

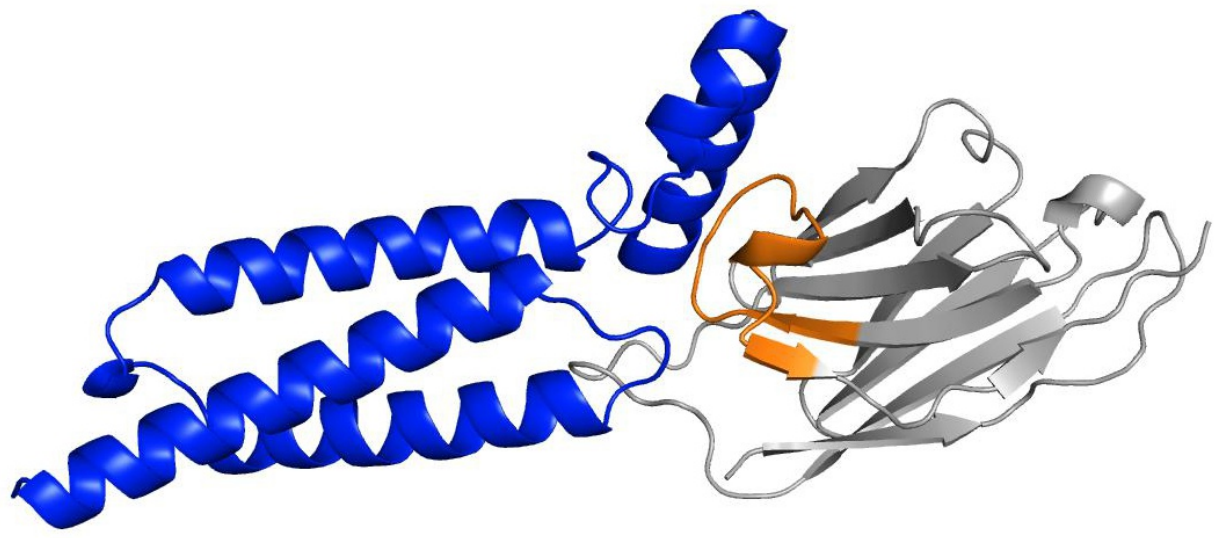

(B) 9GIX, 0 mutations.

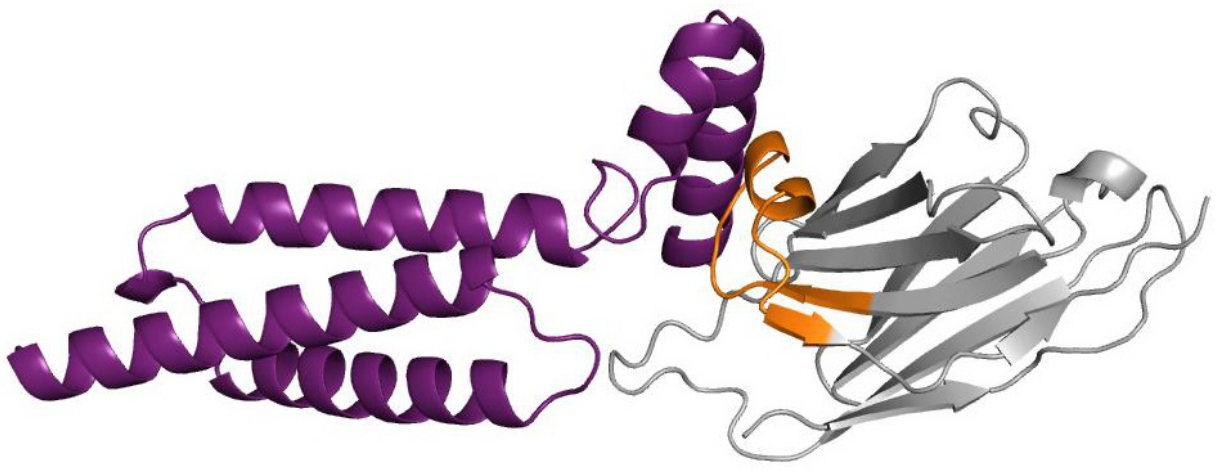

(C) 9GIX, 8 mutations.

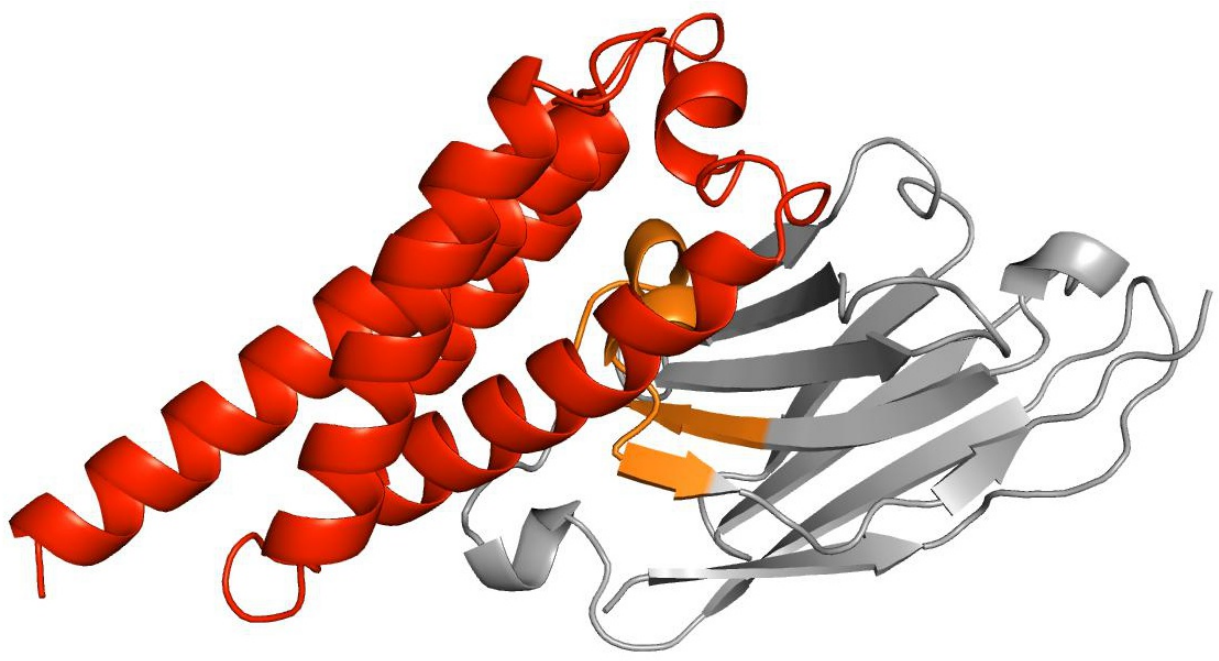

(D) 9GIX, 16 mutations.

Figure S6. (A) Antigen-only Cα-RMSD between each CDR3 alanine-mutated prediction (AF3) and the corresponding experimentally determined antigen structure, versus the number of alanine mutations in the nanobody sequence, one line per antigen. (B–D) The 9GIX complex predicted at 0, 8, and 16 CDR3 alanine mutations, superimposed on the nanobody (grey, CDR3 in orange); the antigen (blue $\rightarrow$ red with increasing mutation count) swings progressively away from its 0-mutation pose, illustrating the strong 9GIX antigen drift seen in (A) even though its nanobody barely moves.

0.6 Details for CD33 camelid immunisation campaign

In Figure S7 we present the ROC AUC for all analysed confidence measures when predicting known binders for CD33 compared to candidate sequences from the immunisation campaign. Note that many measures exhibit an ROC AUC of under 0.5, indicating poor generalisation to this antigen and distribution.

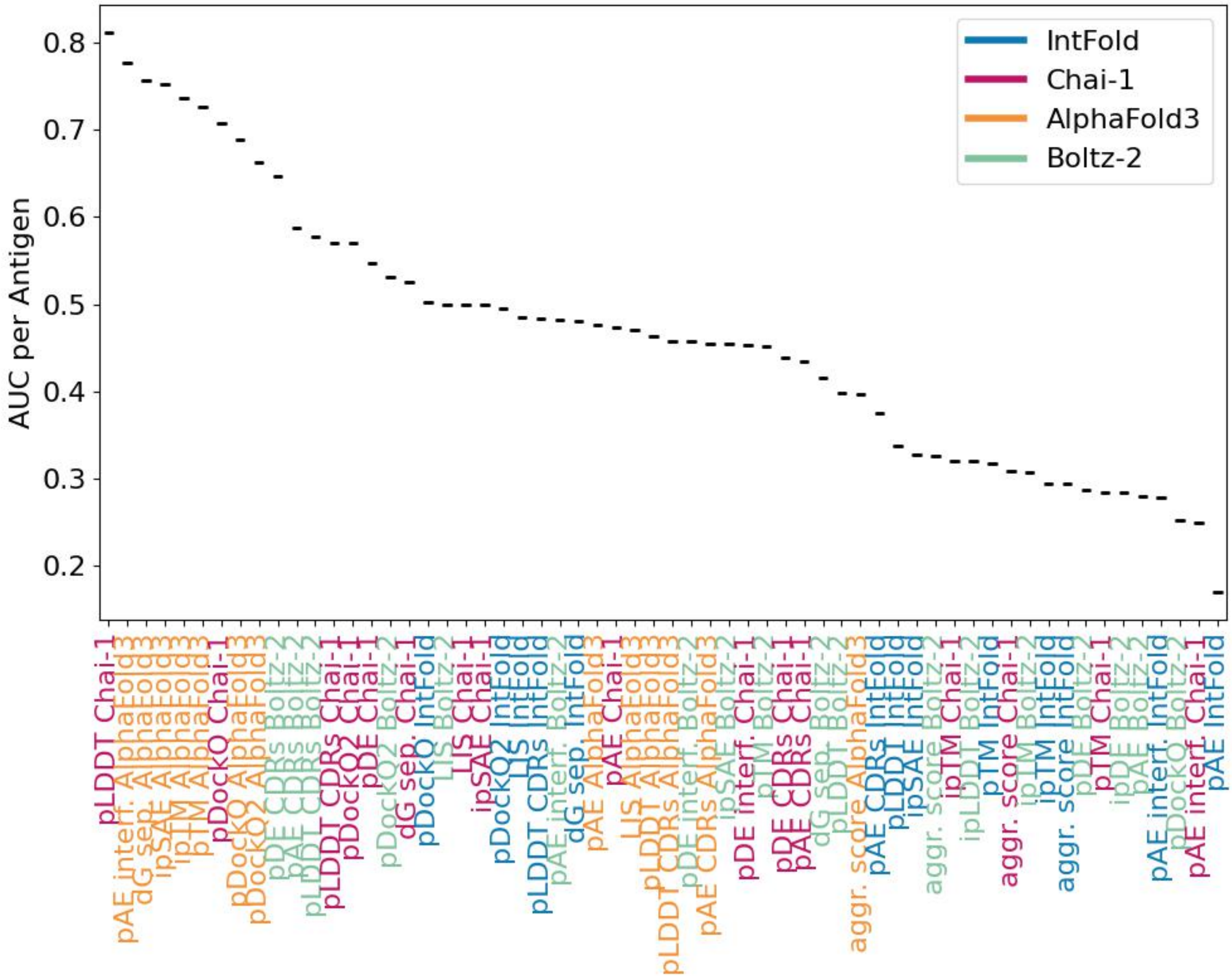

Figure S7. All 60 confidence measures for the prediction of true binders in the camelid immunisation dataset.

0.7 Precision–Recall AUC (PR-AUC)

Our benchmarks are class-imbalanced: each antigen has only a small number of positive sequences relative to the negatives. In true binder detection these positives are the few true binders among many non-binders, while in OOD detection they are the few full-alanine OOD sequences we aim to flag among the many realistic ones. In this regime the ROC AUC we report in the main text remains the appropriate primary metric for our prevalence-invariant screening question (Section 2.3.3), but, being independent of class prevalence, it does not necessarily reflect the precision attainable on the rare positive class at deployment. We therefore additionally report the precision–recall AUC (Precision–Recall Area Under the Curve (PR-AUC)). This complementary prevalence-dependent view quantifies how reliably a measure places the positives at the top of the ranking, computed as the average precision per antigen. We use `sklearn.average_precision_score`, a step-wise estimator that avoids over-optimistic linear interpolation of a trapezoidal precision–recall area, for computations. For each experiment we show, as in the main text, the top confidence measures by median PR-AUC alongside a per-model summary across all measures, as well as the full per-measure distribution over all 17 antigens. Because a precision–recall

analysis is defined with respect to the positive (minority) class, its orientation differs from the ROC AUC reported in the main text: for OOD detection we take the full-alanine sequence as the positive class, which requires inverting the confidence measures (oriented higher = more realistic). The PR-AUC thus quantifies the precision of catching the all-alanine OOD sequences rather than of ranking the realistic sequences above them. This is the natural framing for a precision–recall view, but the inverted orientation should be kept in mind when comparing these values to the ROC AUC.

We provide the PR-AUC figures grouped by experiment. Figure S8 and Figure S9 report true binder detection, Figure S10 and Figure S11 report OOD sequence detection, and Figure S12 and Figure S13 report the CD33 camelid immunisation campaign. For each experiment, the first figure shows the top confidence measures by median PR-AUC alongside a per-model summary, and the second shows the full per-measure distribution across all 17 antigens.

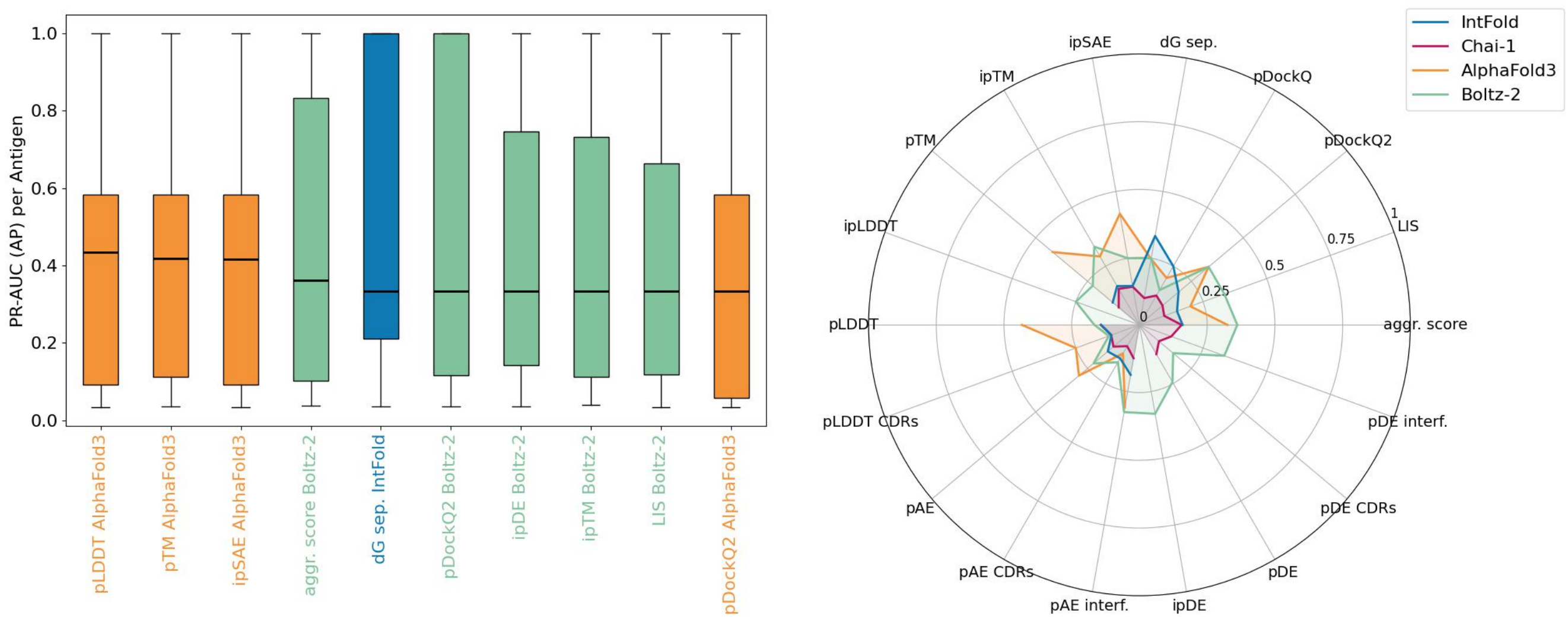

Figure S8. True Binder Detection (PR-AUC). Left: Top 10 confidence measures by median PR-AUC, excluding our CDR and interacting interface specific measures. Right: Median PR-AUC per model across all measures.

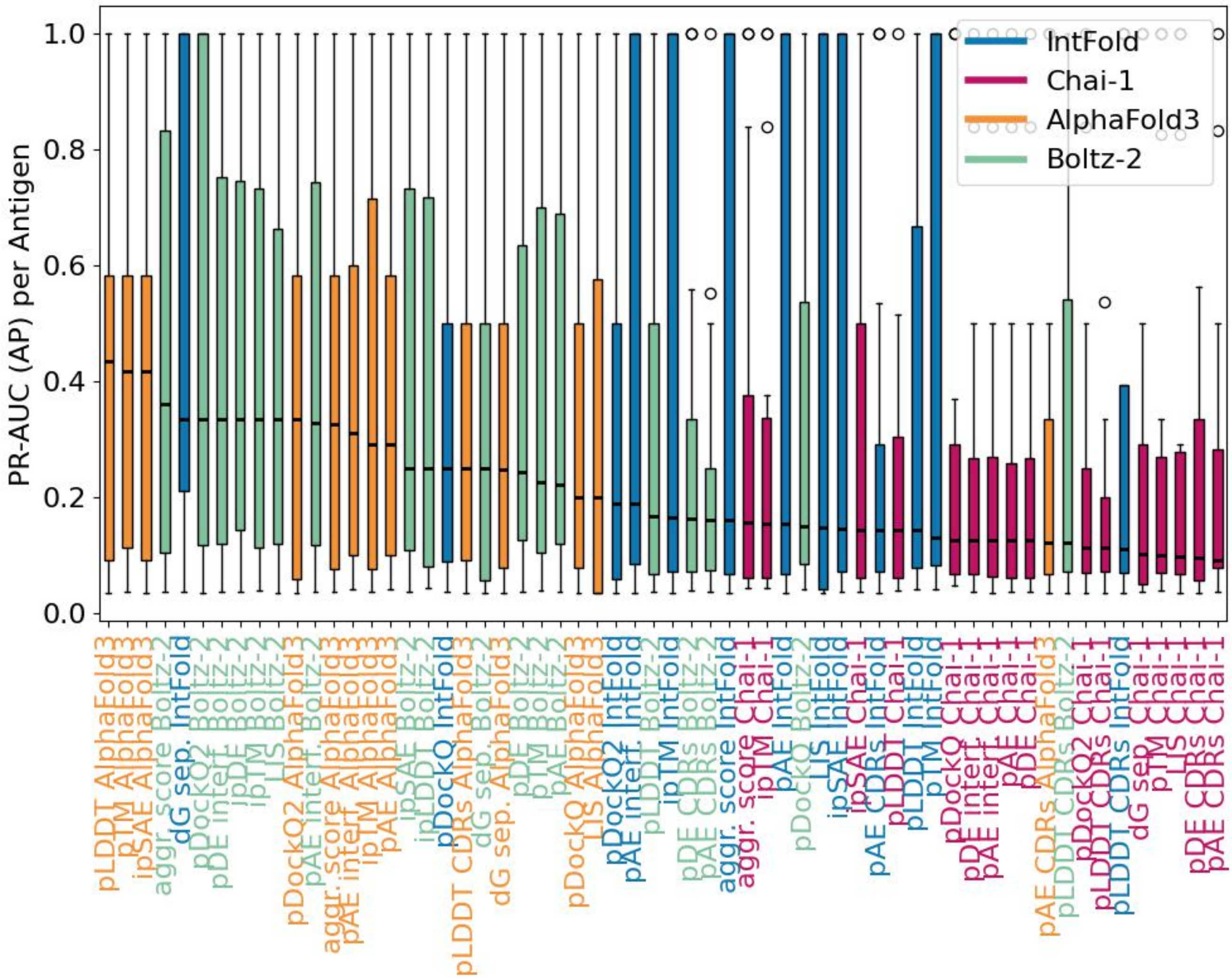

Figure S9. True Binder Detection: Boxplot of PR-AUC for all analysed confidence measures.

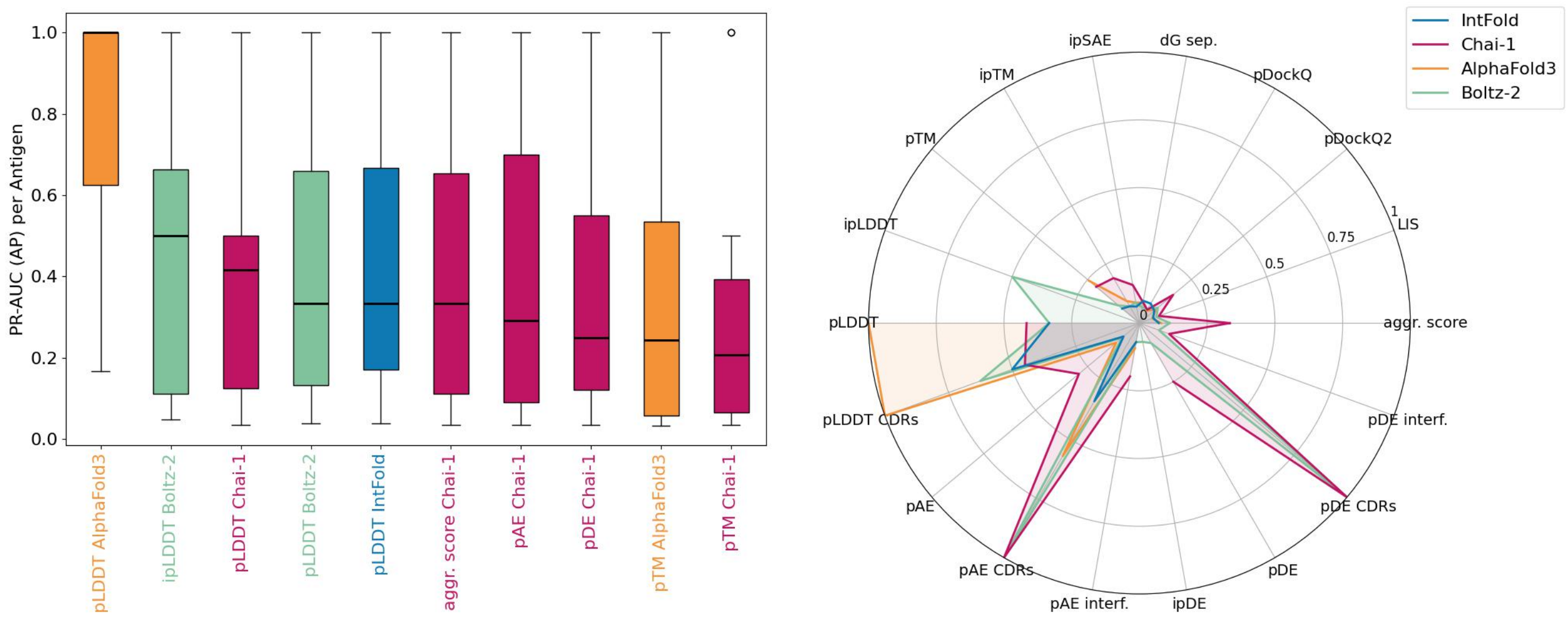

Figure S10. OOD Sequence Detection (PR-AUC). Left: Top 10 confidence measures by median PR-AUC, excluding our CDR and interacting interface specific measures. Right: Median PR-AUC per model across all measures.

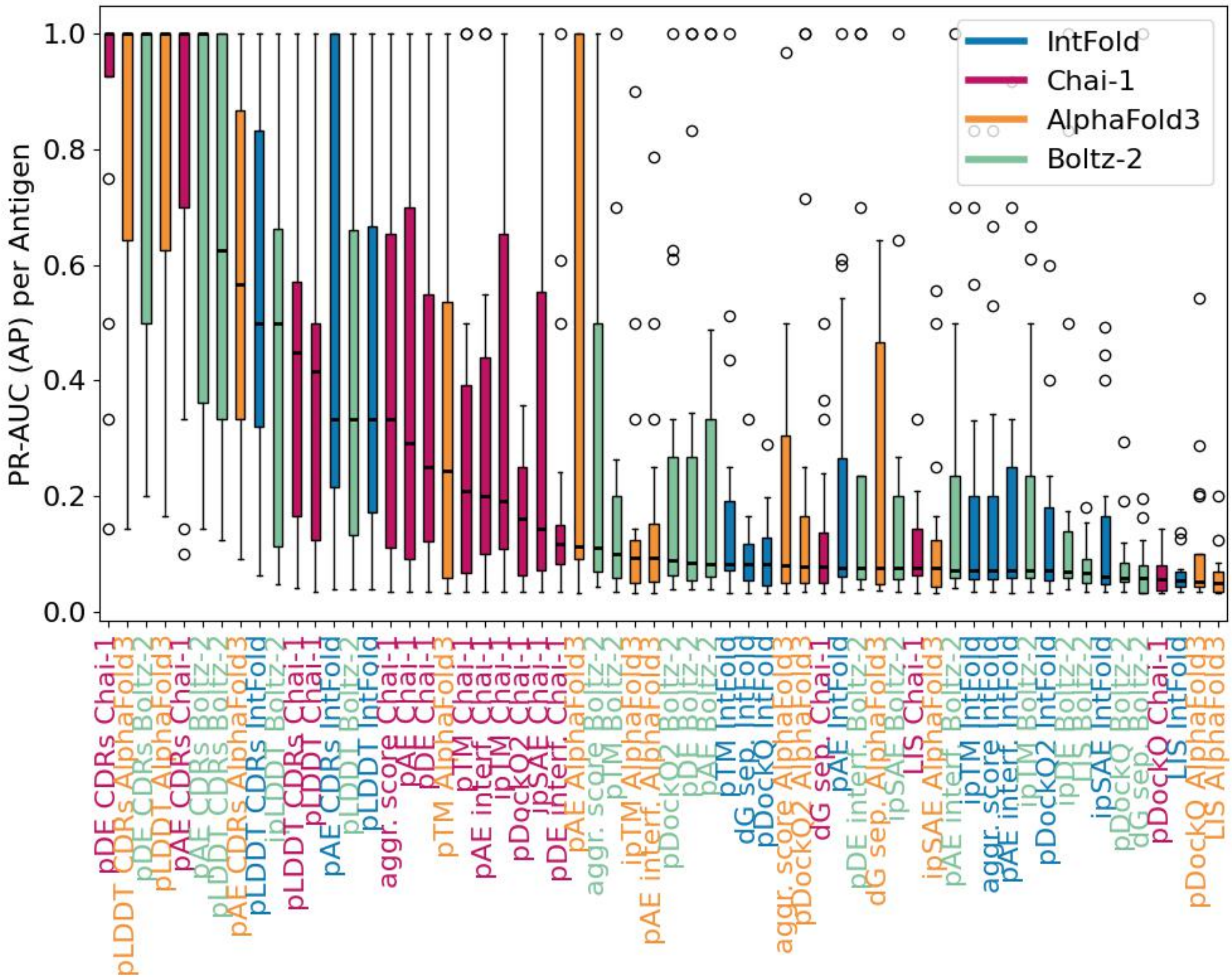

Figure S11. OOD Sequence Detection: Boxplot of PR-AUC for all analysed confidence measures.

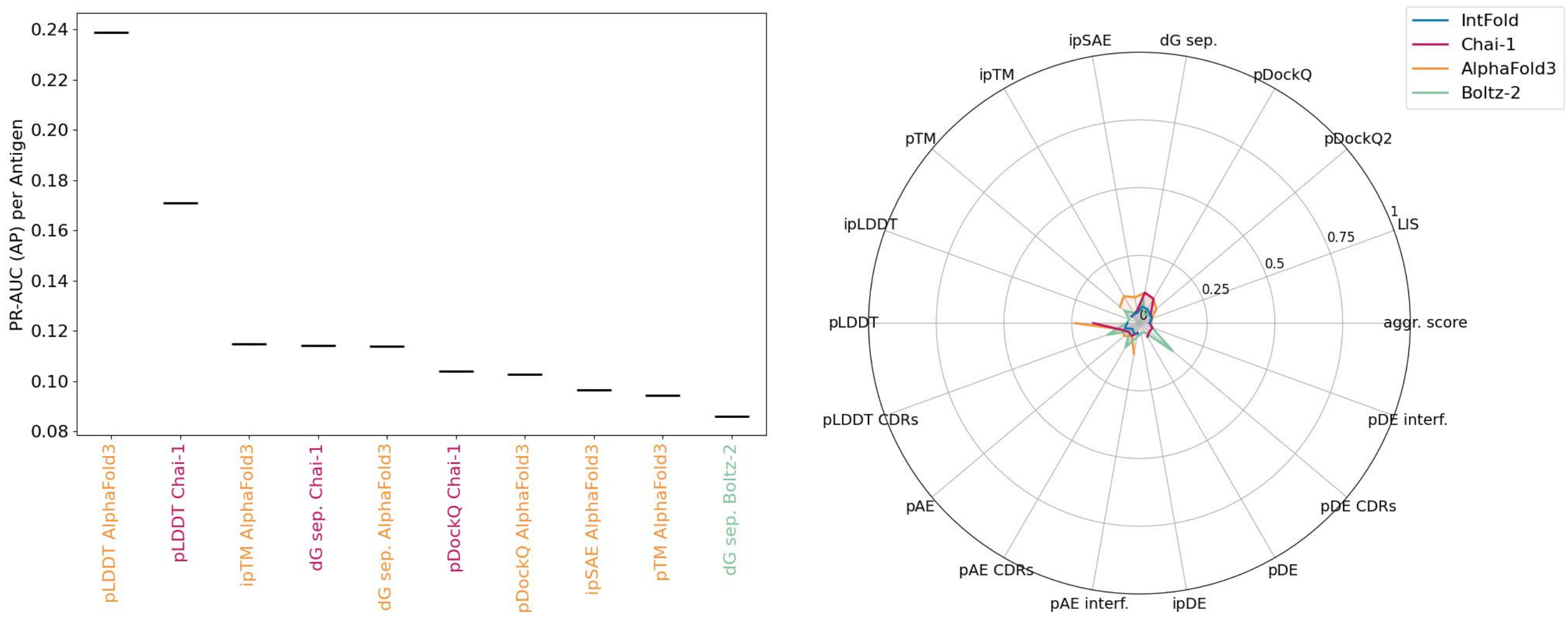

Figure S12. CD33 camelid immunisation campaign (PR-AUC). Left: Top 10 confidence measures by median PR-AUC, excluding our CDR and interacting interface specific measures. Right: Median PR-AUC per model across all measures.

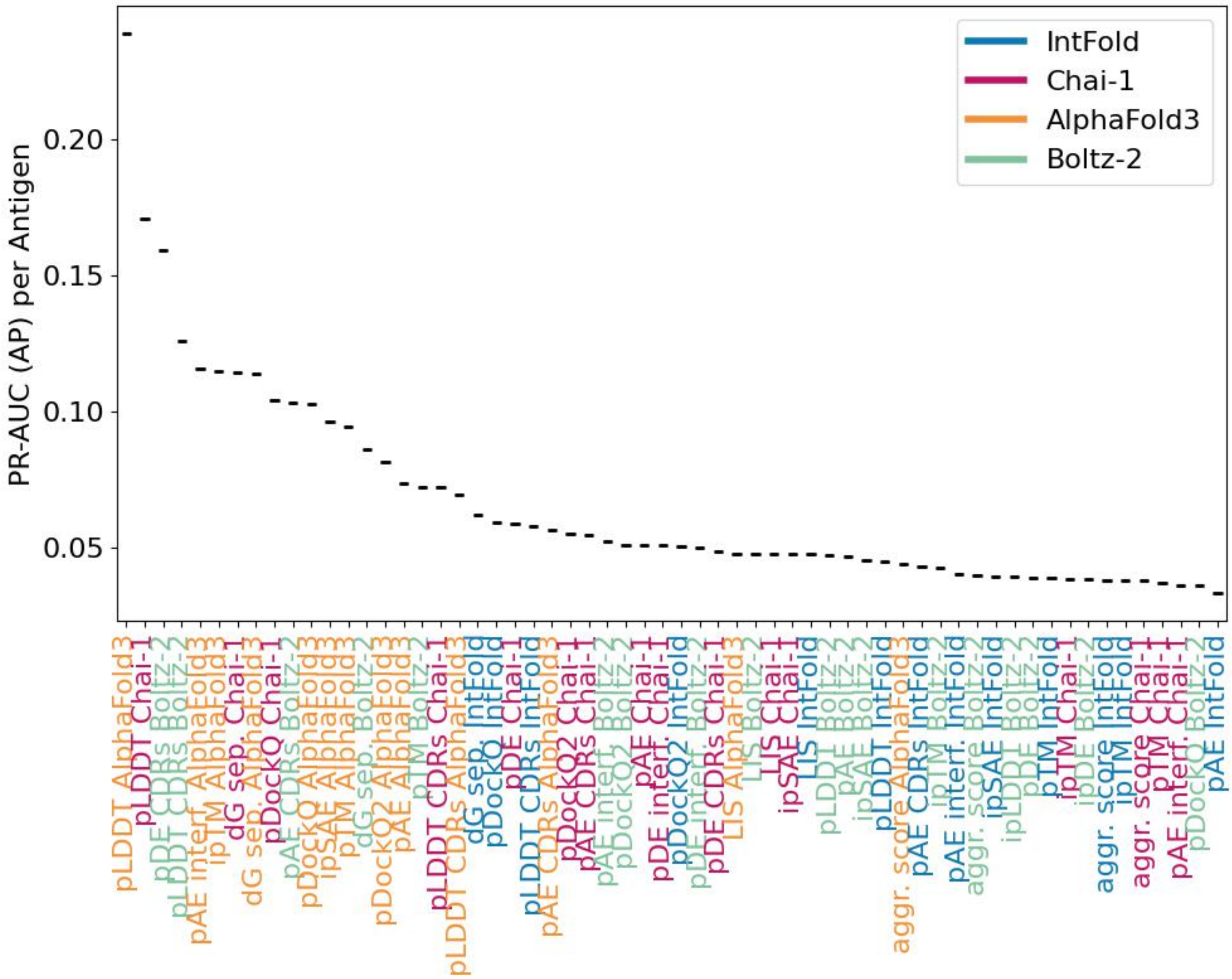

Figure S13. CD33 camelid immunisation campaign: Boxplot of PR-AUC for all analysed confidence measures.

REFERENCES

Dunbar, J. and Deane, C. M. (2016). ANARCI: antigen receptor numbering and receptor classification. *Bioinformatics* 32, 298–300

Mirdita, M., Schütze, K., Moriwaki, Y., Heo, L., Ovchinnikov, S., and Steinegger, M. (2022). ColabFold: making protein folding accessible to all. *Nature Methods* 19, 679–682. doi:10.1038/s41592-022-01488-1

The IntFold Team, Qiao, L., Bai, W., Yan, H., Liu, G., Xi, N., et al. (2025). IntFold: A Controllable Foundation Model for General and Specialized Biomolecular Structure Prediction doi:10.48550/arXiv.2507.02025
